# Modeling of Genome-Wide Polyadenylation Signals in *Xenopus tropicalis*

**DOI:** 10.3389/fgene.2019.00647

**Published:** 2019-07-03

**Authors:** Sheng Zhu, Xiaohui Wu, Hongjuan Fu, Congting Ye, Moliang Chen, Zhihua Jiang, Guoli Ji

**Affiliations:** ^1^Department of Automation, Xiamen University, Xiamen, China; ^2^National Institute for Data Science in Health and Medicine, Xiamen University, Xiamen, China; ^3^Innovation Center for Cell Signaling Network, Xiamen University, Xiamen, China; ^4^Key Laboratory of the Ministry of Education for Coastal and Wetland Ecosystems, College of the Environment and Ecology, Xiamen University, Xiamen, China; ^5^Department of Animal Sciences and Center for Reproductive Biology, Washington State University, Pullman, WA, United States

**Keywords:** alternative polyadenylation, *cis* elements, developmental specificity, bioinformatic analysis, 3′ UTR, maternal-to-zygotic transition

## Abstract

Alternative polyadenylation (APA) is an important post-transcriptional modification event to process messenger RNA (mRNA) for transcriptional termination, transport, and translation. In the present study, we characterized poly(A) signals in *Xenopus tropicalis* using 70,918 highly confident poly(A) sites derived from 16,511 protein-coding genes to understand their roles in the regulation of embryo development and gender difference. We examined potential factors, including the gene length, the number of introns in a gene, and the intron length, that may affect the prevalence of APA. We observed 12 prominent poly(A) signal patterns, which accounted for approximately 92% of total APA sites in *Xenopus tropicalis*. Among them, three patterns are specific to *X. tropicalis*, so they are absent in other animals such as humans or mice. We catalogued APA sites based on their genomic regions and developed a bioinformatics pipeline to identify over-represented signal patterns for each class. Then the schema of *cis* elements for APA sites in each genomic region was proposed. More importantly, APA usage is dramatically dynamic in embryos along five developmental stages and well-coordinated with the maternal-to-zygotic transition event. We used an entropy-based method to identify developmental stage-specific APA sites and identified significant signal patterns around specific sites and constitutive sites. We found that the APA frequency in different genomic regions varies with developmental stages and that those sites located in intron or coding sequence regions contribute most to the dynamics of gene expression during developmental stages. This study deciphers the characteristics and poly(A) signal patterns for both canonical APA sites and non-canonical APA sites across different developmental stages and gender dimorphisms in *X. tropicalis*, providing new insights into the dynamic regulation of distal and proximal APA.

## Introduction

The 3′ untranslated region (3′ UTR) of eukaryotic mRNA plays an important role in the regulation of gene expression (Ji et al., 2007). The addition of an A-stretch to the 3′ end, called polyadenylation [poly(A)], is important for stability, translation, and nuclear export of mRNA (Wu and Brewer, 2012). Genes containing more than one poly(A) site undergo alternative polyadenylation (APA). APA occurs extensively in protein-coding genes and is highly regulated during development (Brutman et al., 2018; Zhang et al., 2018; Zhou et al., 2019). Differential usage of APA sites can be influenced by physiological conditions such as cell growth, differentiation, and development (Di Giammartino et al., 2011; Shi, 2012). Polyadenylation is guided by *cis* elements surrounding the cleavage site [collectively known as poly(A) signals (PAS)] that are recognized by factors in the core polyadenylation process. Numerous studies have recognized a set of poly(A) signals embedded in the pre-mRNA (reviewed in Xing and Li, 2011; Tian and Graber, 2012; Ji et al., 2015) (**Figure S1**). The most predominant PAS in vertebrates is AAUAAA, while it is less common in plants or bacteria (Shen et al., 2008). Several elements are defined as the pivotal PAS motifs located in the 3′ UTR of vertebrate pre-mRNA: AAUAAA and its variants in the positioning element (PE), the U-rich upstream sequence element (USE; 40–80 nt upstream of the cleavage site), and the U/GU downstream sequence element (DSE; 20–40 nt downstream of the cleavage site) (Beaudoing et al., 2000; Tian et al., 2005; Tian and Graber, 2012). In plants, there are three major PAS regions, including far upstream element (FUE), near upstream element (NUE), and the cleavage element (CE) (HM, 1996; Hunt, 1997; Rothnie et al., 2001; Shen et al., 2008). The NUE is an AAUAAA-like element, while FUE is typically U-rich or UG-rich. The yeast PAS motifs generally consist of four elements: “efficiency” element (EE) of which UAUAUA is the core signal, PE of which AAUAAA is the most dominant signal located between the EE and the poly(A) site, and two U-rich elements located upstream and downstream of the cleavage site (Guo and Sherman, 1996a; Guo and Sherman, 1996b; Ozsolak et al., 2010).

Since the late 1990s, the emergence of expressed sequence tag (EST) data has facilitated the large-scale analysis of potential PAS motifs (Gautheret et al., 1998). A number of single-nucleotide variants of AAUAAA have been well studied, including AGUAAA, UAUAAA, CAUAAA, GAUAAA, AAUAUA, AAUACA, AAUAGA, AACUAAA, AAGAAA, and AAUGAA (Beaudoing et al., 2000; Gruber et al., 2016). To date, poly(A) sites of several species have been investigated at a whole genome level, including humans (Beaudoing et al., 2000), chickens, fugu fish, zebrafish (Brockman et al., 2005; Salisbury et al., 2006; Lee et al., 2007; Hutchins et al., 2008), flies (Brockman et al., 2005), mosquitoes (Retelska et al., 2006), nematodes (Hajarnavis et al., 2004), yeasts (Guo and Sherman, 1996a), *Arabidopsis* (Loke et al., 2005), and rice (Shen et al., 2008). *X. tropicalis* is a representative species in the *Xenopus* genus with a diploid genome (Amaya et al., 1998), also known as the tropical clawed frog (Mcdonough, 2014). Its genome has been sequenced (Hellsten et al., 2010), making it an important model organism for genetics, biological, and biomedical studies (Bowes et al., 2008). However, in contrast to the high abundance of APA studies in many other animals and plants, researches that characterize APA sites or poly(A) signals at a genome-wide scale in *X. tropicalis* are scarce. In the early years, studies on *Xenopus* have revealed the role of CPE-binding protein (CPEB) in cytoplasmic polyadenylation (Hake and Richter, 1994) and the effect of downstream sequences of poly(A) sites on the position of 3′ RNA processing (Mason et al., 1986). Until recently, a large number of APA sites from embryos and adults in *X. tropicalis* were collected and analyzed (Zhou et al., 2019). However, there seems to be few reports on the genome-wide analysis of poly(A) signals in *X. tropicalis*, especially on unconventional poly(A) signals in non-3′ UTR regions. With the advancement of sequencing technologies, genome-wide unconventional poly(A) sites continue to accumulate. A recent study (Singh et al., 2018) identified widely expressed intronic poly(A) sites in immune cells and found that these sites are differentially used during B-cell development and can lead to protein truncation. Guo et al. (2016) detected ∼11,000 non-3′ UTR poly(A) sites in *Arabidopsis* and found that the occurrence of these sites was correlated with characteristics of their respective genes, such as alternative splicing of 5′ UTRs, number of introns, and whether an intron has extreme length. However, poly(A) signals of these unconventional poly(A) sites are still poorly understood, and deeper understanding of these *cis* elements would contribute to profiling the panorama of APA mechanism for both 3′ UTR and non-3′ UTR sites. Additionally, how various *cis* elements co-evolve, how they coordinate with the polyadenylation machinery, and what poly(A) signals are specifically used in certain tissues/developmental stages await further characterization. Over the last decade, bioinformatic analyses with high-throughput genomic data have uncovered various poly(A) signals in a wide range of organisms and conditions and suggested that the PAS is more divergent than expected (Beaudoing et al., 2000; Tian and Graber, 2012; Gruber et al., 2016). For instance, Gruber et al. (2016) reanalyzed a large number of 3′ end sequencing (3′ seq) datasets in humans and mice and found 18 poly(A) signals with six novel signals. Common and divergent *cis* elements between *X. tropicalis* and other vertebrate and their effect on polyadenylation efficiency under different conditions need further characterization. The availability of whole genome APA sites in *X. tropicalis* provides opportunities to extend bioinformatic analysis for the in-depth study of *cis* elements that control alternative polyadenylation in *X. tropicalis*.

Here, we developed bioinformatics pipelines to decipher poly(A) signals in *X. tropicalis* using a large poly(A) site dataset compiled from nine developmental stages of *X. tropicalis*. We examined potential factors, including the gene length, the number of introns in a gene, and the intron length, that may affect the prevalence of APA. We classified 3′ UTR poly(A) sites based on their expression levels and identified poly(A) signals according to their polyadenylation efficiency. Then we proposed the schema of poly(A) signals for 3′ UTR sites and identified three patterns that are unique in *X. tropicalis*. More importantly, we classified APA sites based on their proximal genomic regions and identified over-represented and unique signal patterns in each category. Further, we designed distinct schemas of *cis* elements for APA sites in 5′ UTR, coding sequence (CDS) region, and intron. Further, we used an entropy-based method to identify developmental stage-specific APA sites, and we identified significant signal patterns around specific sites and constitutive sites. We found that APA frequency varies across different developmental stages and is also different between female and male. Particularly, APA frequency in different genomic regions varies with developmental stages, and those sites located in intron or CDS regions contribute most to the dynamics of gene expression during developmental stages. This study deciphers the characteristics and poly(A) signal patterns for both canonical 3′ UTR APA sites and non-canonical APA sites across different developmental stages and gender dimorphisms in *X. tropicalis*, providing new insights into the dynamic regulation of distal and proximal APA.

## Materials and Methods

### Identification of Poly(A) Sites in *X. tropicalis*

The data were derived from *X. tropicalis* embryos at five developmental stages (three biological replicates) and adult male and female frogs at two developmental stages (two biological replicates) using the whole transcriptome termini site sequencing (WTTS-Seq) method (Zhou et al., 2016). Initially, 127,914 poly(A) site clusters (PACs) were obtained (Zhou et al., 2019). These PACs were generated by grouping nearby cleavage sites within 24 bp of each other. In order to obtain highly confident poly(A) sites, 33,039 APA sites located in the upstream regions of A-rich stretch (ARS) were filtered out. ARS refers to a site with six or more adenines within 10 nt downstream of the 3′ end cleavage site. To further remove low-quality poly(A) sites, those with AAAA, AGAA, AAGA, or AAAG immediately downstream of the cleavage site (Gruber et al., 2016) or with more than five adenines in succession within 10 nt downstream of the cleavage site were discarded. Finally, 94,875 sites were retained, 70,918 of which were located in protein-coding genes. To study the signal patterns of poly(A) sites, the sequence surrounding each poly(A) site was extracted from the *X. tropicalis* genome (NCBI assembly Xenopus_tropicalis_v9.1).

### Classification of Poly(A) Sites

We adopted a similar strategy reported previously (Legendre and Gautheret, 2003; Hu et al., 2005) to classify poly(A) sites based on their expression levels. Strong sites are those APA sites supported by more than 70% of total reads of the respective gene. Weak sites are the remaining sites in the gene with a strong site. Universal sites are from genes with multiple sites but without a strong site. Unique sites are from genes containing only one poly(A) site. Control sites are random positions in the genome with AAUAAA but without any real poly(A) site within 50 nt around the AAUAAA signal.

### Identification of Over-Represented Poly(A) Signal Patterns

We refer to the method by Beaudoing et al. (2000) to filter over-represented poly(A) patterns. The over-represented patterns around 3′ UTR poly(A) sites were determined by comparing the expected frequency with the real frequency of a pattern in a given signal region. The expected frequency of occurrence was calculated based on the first-order Markov chain (MC) model for a particular poly(A) region of all poly(A) sites. For example, for a given hexamer AAUAAA, its frequency is calculated as follows:

(1) $$f_{e}\left(AAUAAA\right)=\frac{f_{o}\left(AA\right)\times f_{o}\left(AU\right)\times f_{o}\left(UA\right)\times f_{o}\left(AA\right)\times f_{o}\left(AA\right)}{f_{o}\left(A\right)\times f_{o}\left(U\right)\times f_{o}\left(A\right)\times f_{o}\left(A\right)}$$

where *f_o_* (*AA*) is the observed frequency of occurrence of AA. Then the expected number of occurrences [*O_e_*(*kmer*)] can be calculated:

(2) $$O_{e}\left(kmer\right)=\;f_{e}\left(kmer\right)\times \sum_{i=1}^{n}\left(l_{i}-k+1\right)$$

where *kmer* refers to a nucleotide subsequence of length *k*, *n* refers to the total number of sequences to be counted, and *l_i_* refers to the length of the *i*th sequence. However, the self-overlapping increases the second moment (variance), leading to a higher probability of observing occurrence values distant from the average. The estimated variance was corrected for self-overlapping patterns and was introduced in calculating the *z*-score:

(3) $$Z_{oe}\left(kmer\right)=\frac{\left[O_{o}\left(kmer\right)-O_{e}\left(kmer\right)\right]}{SD\left(kmer\right)}$$

where *O_o_*(*kmer*) is the occurrence of *kmer* in a specific region of all poly(A) sites. *O_o_*(*kmer*) is an expected occurrence and was calculated based on the MC model of a specific poly(A) region of all poly(A) sites. *SD* (*kmer*) is the standard deviation of the *kmer* distribution.

Probabilities were computed assuming a cumulative binomial distribution (Press et al., 1988). The spatial distribution of well-expressed hexamer has good “clustering” characteristics. The smaller the standard deviation of the distribution is, the more concentrated the hexamer distributes. Therefore, the *p*-value and standard deviation were jointly considered to select the most significant motifs. Since a pattern can vary along the location scale, some hexamers may be partially overlapped with each other, for instance, AAAUAA, AUAAAA, and AAUAAA. In order to reduce the statistical error rates and repeat rates, before we count the next most frequent hexamer, we will remove the sequence covered by the current most frequent hexamer from the dataset once selected. Since the occurrence of different hexamers may be related, this method not only ensures the occurrence of repeated hexamers but also ensures that the correlations among the selected hexamers are low. However, when the sequences containing a hexamer were removed from the dataset, the incidence of other hexamers was weakened correspondingly, and the standard deviation of the hexamer position was increased, thereby reducing the judgment. Therefore, we chose to use the standard deviation obtained from the initial dataset as the basis for judgment. Also, considering the spatial distribution of these sites at −34 nt to −13 nt, the average position of each motif in 20 nt was calculated, and the standard deviation (SD) around the mean was used as a measure of the degree of dispersion. Dispersed motifs with SD > 7 nt were unlikely to form a polyadenylation signal, which were not considered in this study.

### Identification of Significant Poly(A) Signals

We used the method described by Hu et al. (2005) to identify significant poly(A) signals. After identifying strong and weak APA sites, we divided each sequence into five regions according to the poly(A) signal models discovered in mammals and plants (**Figure S1**).

In Equation (3), we introduced the *z*-score to calculate the difference between the observed and expected occurrences (*z_oe_*). Considering the effects of different types of sites, we not only calculated *z_oe_* for each hexamer for a particular region but also calculated the difference of the frequency of occurrence (*z_sw_*) between the strong and weak poly(A) sites in each signal region (Eq. 4).

(4) $$Z_{sw}\left(kmer\right)=\frac{O_{s}\left(kmer\right)-O_{w}\left(kmer\right)}{\sqrt{\left(\frac{1}{N_{s}}+\frac{1}{N_{w}}\right)\times p\times \left(1-p\right)}}$$

where *kmer* refers to the subsequences (of length *k*) from the poly(A) dataset sequences. *O_s_* (*kmer*) and *O_w_* (*kmer*) are the frequency of occurrence of the *kmer* in strong and weak poly(A) regions, respectively. *N_s_* and *N_w_* are the total number of all *kmers* in a specific region of strong poly(A) sites and weak poly(A) sites, respectively. The probability of the given *kmer*, *p*, is calculated as follows:

(5) $$p=\frac{f_{s}\left(kmer\right)\times O_{s}+f_{w}\left(kmer\right)\times O_{w}}{\left(O_{s}+O_{w}\right)}$$

To obtain significant hexamers (*k* = 6), we combined these two *z*-scores (*z_oe_* and *z_sw_*) and selected a set of cutoff values ([Cz_sw_, Cz_oe_]) for screening hexamers. Next, we grouped the selected hexamers based on their mutual distance, and we clustered them based on their dissimilarity distance. In this study, hexamers were grouped based on a cutoff of 2.6. And hexamers in the same group were further aligned by ClustalW (Higgins and Sharp, 1988). Finally, the aligned hexamers were used to generate sequence logos by the Web Logo tool (Crooks et al., 2004) and consensus sequences.

### Identification of Developmental Stage-Specific Poly(A) Sites

We adopted an outlier detection method called ROKU (Kadota et al., 2006; Ji et al., 2018) to identify developmental stage-specific poly(A) sites. The conventional information entropy (H) and the adjusted information entropy (modeH) were calculated for each poly(A) site. The information entropy is denoted as

(6) $$H=-\sum_{\text{k=1}}^{n}p_{k}log_{2}\left(p_{k}\right)$$

where *n* is the total number of all developmental periods (*n* = 9) and *p_k_* is the relative expression level of a poly(A) site in a developmental period *k*. *p_k_* is calculated as follows:

(7) $$p_{k}=\frac{x_{k}}{\sum_{i=1}^{n}x_{k}}.$$

*H* ranges from 0 to log_2_ (*n*). According to the *H* values, we defined two types of poly(A) sites, including the poly(A) site highly expressed in a few developmental periods (specific site, *H* is close to 0) and the one uniformly expressed in all development periods [constitutive site, H is close to log_2_ (*n*)]. We used the original vector *x* of a gene and processed expression vector *x*′ to calculate the information entropy (H and modeH) as a measure of the overall development periods. The processed expression vector *x*′ for period *k* is defined as follows:

(8) $${x^{\prime }}_{k}=\left|x_{k}-T_{bw}\right|$$

where *T_bw_* is the one-step Tukey biweight, a popular statistic robust against outliers (Hubbell et al., 2002). After we calculated the values of the two kinds of information entropy for all poly(A) sites, we filtered specific sites and constitutive sites separately by two cutoff values.

### Significance Analysis

The Pearson correlation was calculated using the rcorr function in the R package Hmisc, and the statistical significance of the correlation is obtained by cor.test function in the R package stats. If the *p*-value from the cor.test function is smaller than a given cutoff (e.g., 0.05), then the two variables are significantly correlated. For the hypothesis testing conducted in this study (e.g., *t*-test and correlation test), underlying assumptions such as normality of data were examined and guaranteed to be satisfied.

## Results

### Genome-Wide Distribution of Poly(A) Sites in *X. tropicalis*

**Figure 1:**
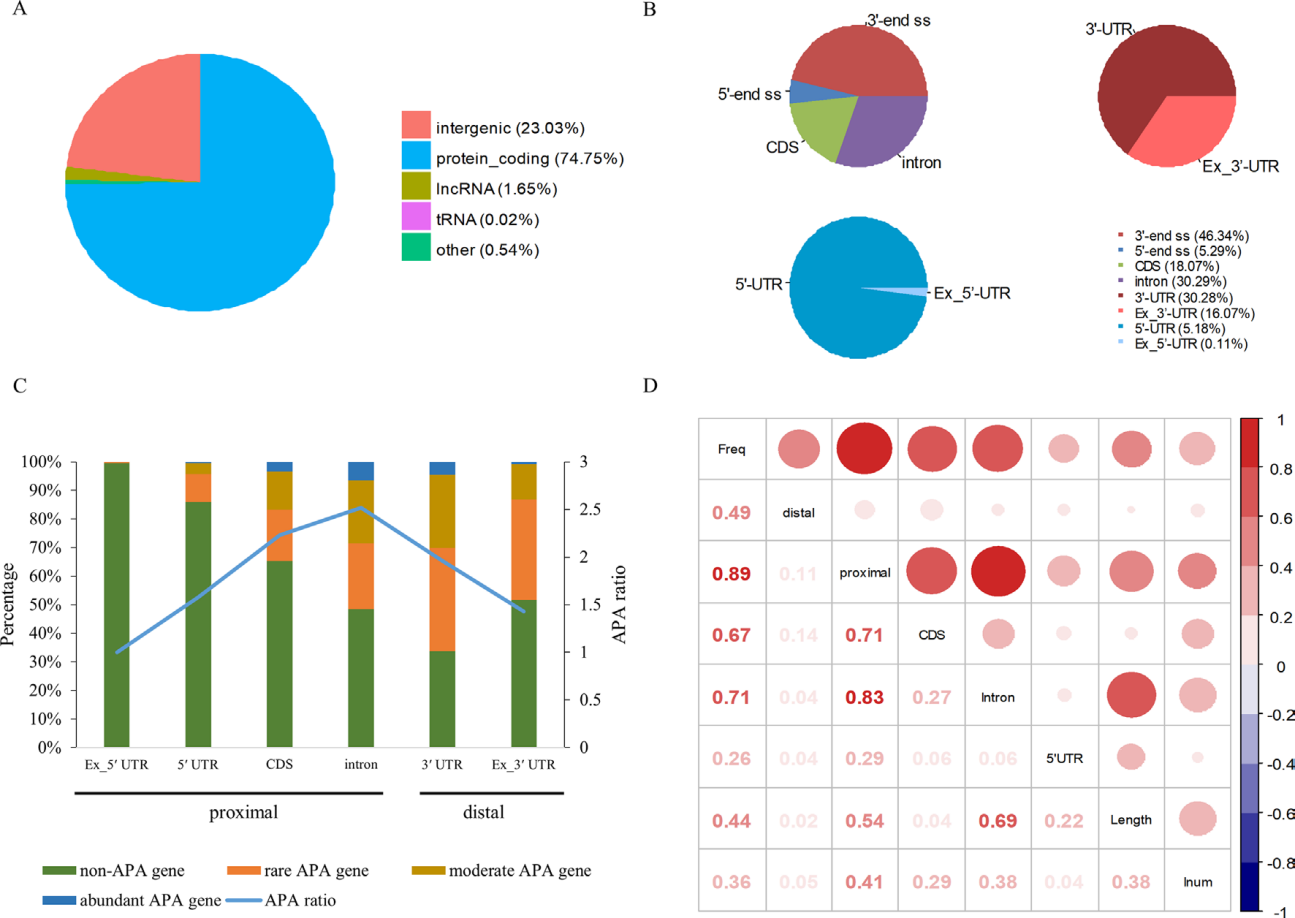
Genomic distribution of poly(A) sites in *X. tropicalis*. **(A)** Distribution of different types of genes. Protein-coding gene accounts for more than 97% of annotated genes. **(B)** Distribution of APA sites in different locations. “3′ end ss” refers to the 3′ UTR and the extended region of 3′ UTR. “5′ end ss” refers to the 5′ UTR and the extended region of 5′ UTR. “Ex_3′ UTR” refers to the extended region of 3′ UTR. “Ex_5′ UTR” refers to the extended region of 5′ UTR. **(C)** Distribution of APA frequencies in different genomic regions. The left *y*-axis denotes the percentage of respective genes with the poly(A) site(s) in the specific region. The right *y*-axis denotes the APA ratio, which is the ratio between the number of APA sites in the specific region and the number of genes these APA sites are located in. Proximal sites are defined as poly(A) sites located in non-3′ UTR regions, while distal sites are those in 3′ UTR or extended 3′ UTR regions. “non-APA gene” refers to no APA event in the gene. “rare APA gene” refers to one APA site per gene. “moderate APA gene” refers to two to four APA sites per gene. “abundant APA gene” refers to more than four APA sites per gene. “APA ratio” refers to the average number of APA per gene (APA site number/APA gene number). **(D)** The relationship among the APA frequency, the gene length, the number of introns, etc. “Freq” represents the frequency of APA sites in the gene; “Length” represents the length of a gene; “Inum” represents the number of introns; “5′ UTR,” “CDS,” and “Intron” represent the frequency of APA sites in the respective genomic regions, respectively. “proximal” represents the frequency of APA sites in CDS, intron, or 5′ UTR regions. “distal” represents the frequency of APA sites in 3′ UTR.

Among 94,875 highly confident APA sites used in the present study, 73,021 (77%) were assigned to currently annotated genes (NCBI *X. tropicalis* genome v9.1), while 21,854 (23%) were located in the intergenic regions (**Figure 1A** and **Table S1**). Of the 73,021 annotated APA sites, 70,918 (97.1%) were associated with 16,511 protein-coding genes. These sites were then divided into different categories according to their genomic locations, such as 3′ UTR, 5′ UTR, coding region (CDS), and intron. Generally, APA sites are abundantly located in 3′ UTR and the extended 3′ UTR regions (**Figure 1B** and **Table S2**). Specifically, 16.1% of poly(A) sites were found in the downstream region of the 3′-end of genes, and about half of these sites occurred within a vicinity of 500 nt (**Table S2**). This observation indicates that the gene annotation in *X. tropicalis* is likely inaccurate and/or incomplete. In addition, the number of sites in introns is equivalent to the number of sites in 3′ UTRs.

APA is widespread in *X. tropicalis* in that more than 75% of genes have multiple poly(A) sites and 34.5% of genes contain five or more poly(A) sites (**Table S3**). Next, we attempted to examine potential factors, including the gene length and the number of introns in a gene, that may affect the frequency of APA events in a gene (herein called APA frequency). To this end, first we classified all expressed genes into four groups: abundant APA gene (five or more APA sites per gene), moderate APA gene (two to four APA sites per gene), rare APA gene (one APA site per gene), and non-APA gene (no APA in the gene). We then compared the distribution of APA sites in different genomic regions among the four groups of APA genes. Apparently, the APA frequency varies greatly across different regions, and only the percentage of APA genes with APA sites in intron and 3′ UTR exceeds 50% (**Figure 1C**). Although a comparable number of APA sites were found in 3′ UTR and intron, the proportion of APA genes with 3′ UTR sites is higher, while the APA ratio (APA site number/APA gene number) is lower (**Figure 1C**). This result suggested that APA frequency is related to its location and that the APA ratio in CDS and intron is generally higher than that in other regions. Overall, there are multiple proximal APA sites on a single gene, most of which have only a single distal APA site. Moreover, the correlation analysis between APA frequency and gene length or the number of introns was conducted (**Figure 1D**). The APA frequency of a gene showed a moderately positive correlation with both the gene length (*r* = 0.44, *p*-value < 2 × 10^−16^) and the number of introns (*r* = 0.36, *p*-value < 2 × 10^−16^). In addition, the correlation between the overall gene APA frequency and proximal APA frequency (*r* = 0.89, *p*-value < 2 × 10^−16^) is much higher than that between the overall gene APA frequency and distal APA frequency (*r* = 0.49, *p*-value < 2 × 10^−16^), which indicates that the frequency of proximal APA contributes more to the APA frequency of the entire gene and exhibits a stronger correlation with the gene length and the number of introns. Moreover, the correlation between the APA frequency in CDS and the overall gene APA frequency (0.67) is similar to that between the APA frequency in intron and the overall gene APA frequency (0.71). But the correlation between the gene length and APA frequency in intron (0.69) is much higher than that between the gene length and APA frequency in CDS (0.04). Taken together, the number of introns in a gene and the gene length have a positive correlation with the APA frequency and may influence the prevalence of APA.

### 3′ UTR Polyadenylation Signals in X UTR Polyadenylation Signals in *X. tropicalis*

**Figure 2:**
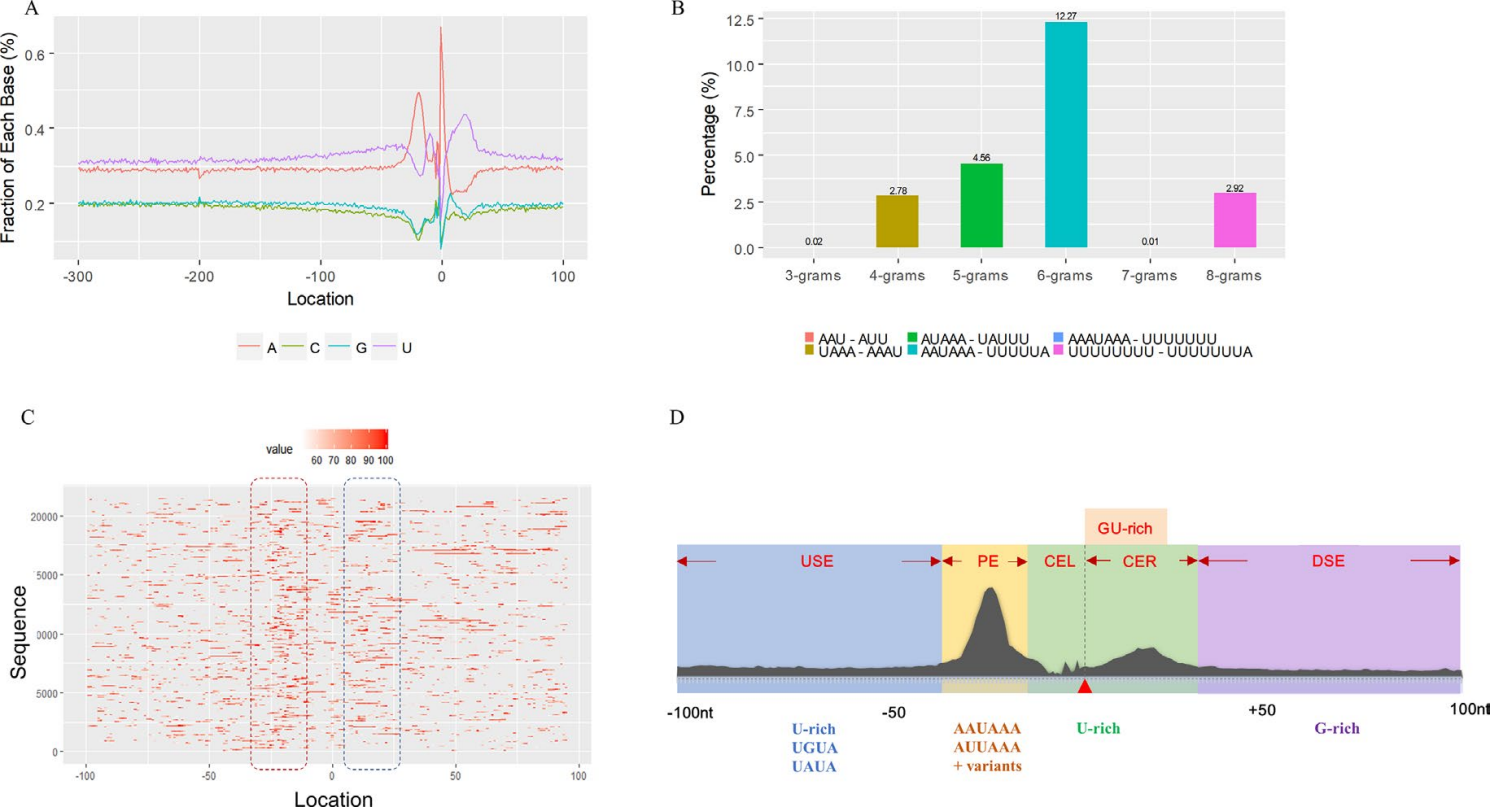
Characteristics of poly(A) signals. **(A)** Nucleotide profiles surrounding poly(A) sites. **(B)** The difference of frequency between the highest signal and the second highest signal (displayed in the legend) with different pattern sizes. **(C)** Top 50 hexamers visual alignment as in the sequence graphics view. Each sequence is present as a single pixel on a horizontal line, and the bright spot represents each occurrence of the signal patterns with respect to their locations on each sequence. The pattern is ranked according to the total frequency that appears in the dataset. The higher the ranking is, the brighter the point is represented. The continuous vertical band of lines from top to bottom indicates the common locations of the signal element. AAUAAA (brightest point) mainly appears around −30 nt to −10 nt (the red dashed box), and the signal aggregation was also observed around +20 nt (the blue dashed box). **(D)** Schematic of *cis* elements for poly(A) sites in* X. tropicalis*. Five regions were determined based on the nucleotide composition profile and the signal analysis. The GU-rich element is overlapped with the downstream U-rich element.

We used a total of 21,472 APA sites located in 3′ UTR for investigating their poly(A) signal landscape (**Table S2**; see Materials and Methods). To identify poly(A) signals near 3′-ends, we extracted upstream 300 nt and downstream 100 nt sequence surrounding each poly(A) site to profile nucleotide distributions (**Figure 2A**). Apparently, the single-nucleotide profile in *X. tropicalis* is similar to that reported in other vertebrates (Legendre and Gautheret, 2003; Lee et al., 2007). The distributions of A and U are clearly distinct, where high U appears in the range of −100 nt to −29 nt, followed by high A in the range of −28 nt to −14 nt. The cleavage site CS (−2, −1) has an apparent YA dinucleotide (Y = U or C) pattern, where the content of A reaches a peak. There is also an obvious U-rich area downstream of CS.

Next, we analyzed the occurrence frequency of poly(A) signals in details, including the dominant hexamer AAUAAA and its variants that bind weakly to the cleavage and polyadenylation specificity factor (CPSF) (**Table S4**). Particularly, AAUAAA was found to be associated with up to 8,390 (47.7%) poly(A) sites. To determine significant *k*-grams for subsequent analysis, we compared the difference in frequency of different *k*-grams and finally decided to use the hexamer (*k* = 6) because the frequency difference between the top two most dominant hexamers is the largest (**Figure 2B**; 12.3%; AAUAAA–UUUUUA). Next, we investigated the potential location of poly(A) signals using the top 50 hexamers with the most frequency of occurrence. Apparently, hexamers are most likely located in the range of −35 nt to −10 nt upstream of poly(A) sites (**Figure 2C**). Besides, aggregation of signals was also observed around 20 nt downstream of poly(A) sites, indicating an auxiliary signal downstream of the CS.

It has been reported that the absence of certain *cis* elements tends to reduce polyadenylation efficiency rather than terminate the process (Tian and Graber, 2012). Next, we examined potential poly(A) signals associated with different levels of poly(A) site usage. We divided 3′ UTR poly(A) sites into four categories according to their polyadenylation efficiency: strong sites (2,449), weak sites (4,026), universal sites (5,718), and unique sites (5,385) (Materials and Methods). Besides, 4,276 control sites also with AAUAAA in the upstream region were used as the reference. Sequences of upstream sequence elements and downstream sequence elements (**Figure S1**) flanking these sites were extracted for the subsequent analysis.

In order to measure the correlation between polyadenylation efficiency and nucleotide biases, the nucleotide distribution of poly(A) sites in each of the five categories was profiled (**Figure S2**). An obvious base content variation was observed in the region flanking true poly(A) sites, while no variation was observed in control sites. This indicates that the base composition of this region distinguishes the true poly(A) sites from randomly occurring sites. Moreover, strong sites and unique sites are significantly more U-rich than other types of sites in the region flanking true poly(A) sites. The base composition of strong sites and unique sites is similar, and that of the weak sites and universal sites is also similar. The difference of U content in the downstream region of CS between strong and weak sites is statistically significant (**Table S5**; *t*-test *p*-value = 3 × 10^−5^), indicating that the downstream of CS may be associated with polyadenylation efficiency. A significant difference of U content between weak sites and control sites was also observed (*t*-test *p*-value = 4 × 10^−20^), suggesting that the U composition variation is sufficient to distinguish true sites from false sites. This result indicates that there may be some U-rich elements in the downstream of CS that can act as a recognition factor for effectively identifying poly(A) sites with higher usage. Compared with the downstream region, the difference of U content is smaller in the upstream region (**Table S6**; *p*-value = 0.08 for strong sites vs. weak sites; *p*-value = 3 × 10^−5^ for weak sites vs. control sites). In contrast to the U content, a larger difference was observed for A, C, and G contents of strong and weak sites in the upstream of CS (**Table S7**; 0.02 for strong sites vs. weak sites; 9 × 10^−10^ for weak sites vs. control sites). Overall, the difference of U composition flanking both sides of poly(A) signals contributes to distinguishing between true and false sites and provides clues for discovering significant *cis* elements in 3′ UTR APA sites with different expression levels.

### Schema of Polyadenylation Signals of Distal APA Sites

Several poly(A) signal elements in animals (Legendre and Gautheret, 2003), such as AAUAAA with its variants and U-rich elements, have also been found in *X. tropicalis* (**Table S4**, **Figures S3** and **2A**). Here, we attempted to construct a comprehensive scenario for poly(A) signals of 3′ UTR sites (also referred to as distal APA sites in this study) in *X. tropicalis*, jointly considering poly(A) signals that have been discovered in both mammals and plants (**Figure S1**). First, we determined potential locations of poly(A) signals by scanning all hexamers, and we profiled the distribution of top 50 hexamers with the highest frequency (**Figure S3** and **Table S4**). Based on nucleotide composition profiles and the signal aggregation analysis, the poly(A) signal regions (relative to CS, −1 position) are defined as follows: USE: −100 ∼ −35; PE: −34 ∼ −13; CEL: −12 ∼ −3; CER: +1 ∼ +33; and DSE: +34 ∼ +100 (**Figure 2D**).

We extracted sequences surrounding poly(A) sites and segmented each sequence into five regions according to the signal model to identify corresponding signal patterns (**Figure 2D**). The frequency of occurrence of each signal pattern in each region around the poly(A) site was calculated, and *z*-score was used to measure the standard deviation of each pattern from the expected value based on the Markov chain model (Helden et al., 2000). For each specific region, a cutoff value was defined based on the *z*-score distribution (*p*-value < 0.01). Selected hexamers are then clustered according to their sequence similarity. Similar hexamers in the same cluster were used to generate a sequence logo. Finally, 15 *cis* elements were identified in the above five regions (**Table 1**).

**Table 1:** *Cis* elements of poly(A) sites in *X. tropicalis*.

Region	Sequence logo	Name	No. of hexamers	Top three hexamers
−100/−35	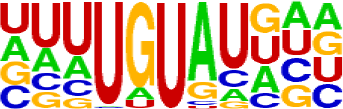	USE.1	32	UUUUAU, UUUUGU, UUUGUA
	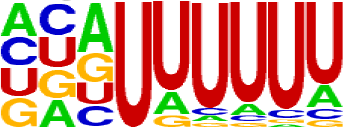	USE.2	25	UUUUUU, UUUUUA, UAUUUU
	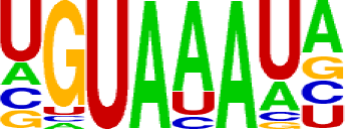	USE.3	6	AAAUGU, AAAUAU, AAUGUU
	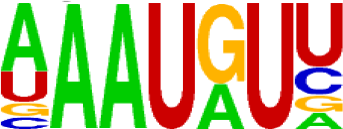	USE.4	5	UGUAAA, UAAAUA, GUAUAU
−34/−13	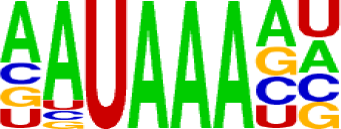	PE.1	13	AAUAAA, AUAAAA, AUAAAU
	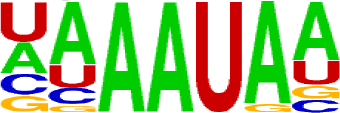	PE.2	19	AAAUAA, UAAAUA, UAAUAA
	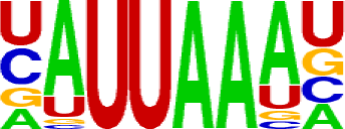	PE.3	5	AUUAAA, UUAAAU, UAUUAA
−12/−3	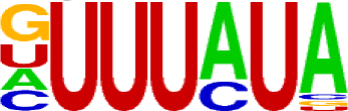	CEL.1	3	UUUAUA, UUUCUA, GUUUAU
	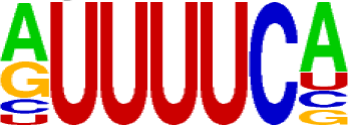	CEL.2	3	UUUUCA, AUUUUC, GUUUUCs
+1/+33	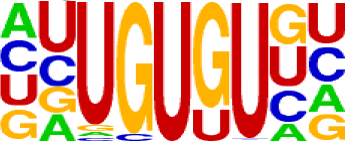	CER.1	15	UUGUUU, UGUGUU, UUGUGU
	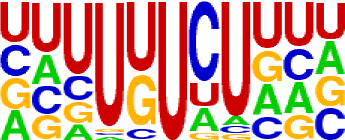	CER.2	30	UUUUUA, UUUUAU, UUUUGU
	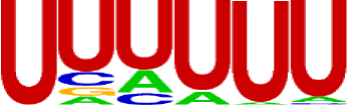	CER.3	14	UUUUUU, UUUAUU, UGUUUU
+34/+100	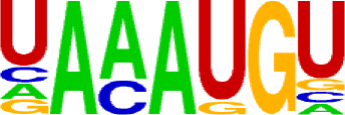	DSE.1	4	AAAUGU, UAAAUG, ACAUGU
	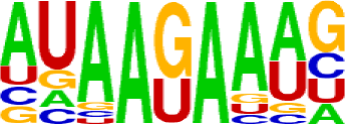	DSE.2	3	AAGAAA, AUAAUA, AGAAUG
	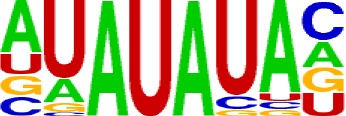	DSE.3	7	UAUAUA, AUAUAU, AAUAUA

**Figure 3:**
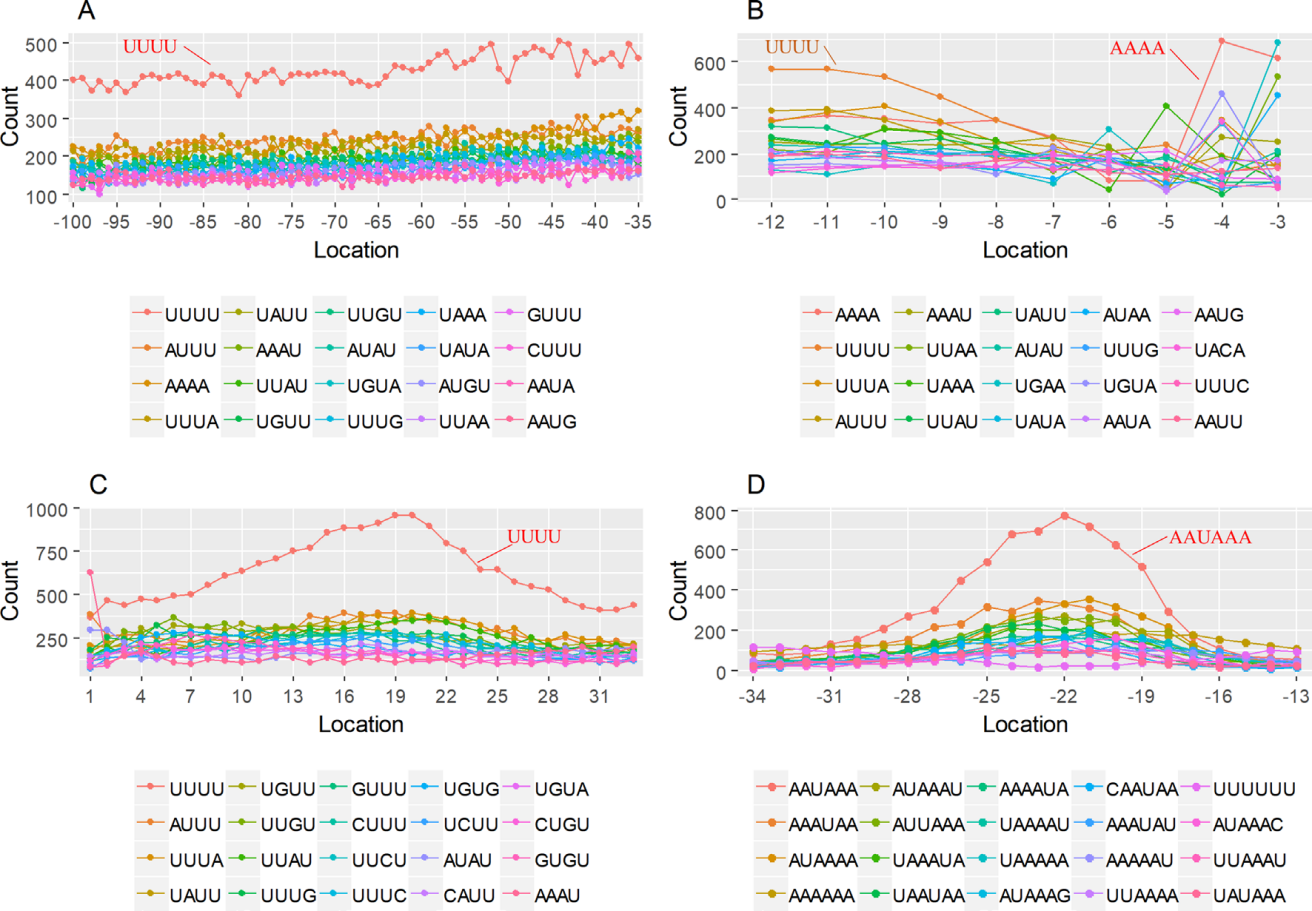
Top-ranked patterns in different poly(A) signal elements. **(A)** Top 20 4-nt patterns in USE according to the occurrence number in the upstream region of poly(A) site (−34 to −100 bp). **(B)** Top 20 4-nt patterns in CEL according to the occurrence number in the upstream region of poly(A) site (−3 to −12 bp). **(C)** Top 20 4-nt patterns in CER according to the occurrence number in the downstream region of poly(A) site (2 to 34 bp). **(D)** Top 20 4-nt patterns in PE region according to the occurrence number in the downstream region of poly(A) site (−13 to −34 bp).

The previous study has revealed an upstream UGUA element as a general poly(A) signal for some human genes without canonical AAUAAA (Venkataraman et al., 2005; Tian and Graber, 2012). Here, we attempted to examine whether there are over-represented tetramers in the USE region. Indeed, UA-rich elements appeared more frequently in USE by counting the high-frequency motifs (**Figure 3A**). Particularly, the occurrence rate of UGUA in this 200 nt sequences set is 80.97%, with an incidence of about 47% in the USE region (**Table S5**). Compared with other U-rich regions with similar nucleotide composition (downstream of the cleavage site, +1 to +96), UGUA was only found in 34% of the CER sequences. This result indicates that UGUA is exclusively over-represented in the upstream region of the poly(A) site (USE). CEL, a signal element spanning a small interval, has been found evolutionarily conserved in mammals, plants, and yeast (Graber et al., 1999). U-rich tetramers were found over-represented in CEL (**Figure 3B**). From the above analysis, it was observed that the CEL signal is more significant in upstream of strong sites than that of weak sites (**Figure S2**), suggesting that CEL is related to APA site usage. Previous studies in human poly(A) signals revealed that the CER contains various U-rich and GU-rich elements, and the GU-rich element is usually closer to the poly(A) site than the U-rich element (Salisbury et al., 2006). In *X. tropicalis*, the frequency of both U-rich and GU-rich components is very high in CER (**Figure 3C**); the closer the GU-rich element is to CS, the higher the frequency is. GU-rich elements, such as UGUG, UGUU, UGAA, and UGUA, are distributed approximately 30 nt downstream of CS and are clearly detected in CER.

AAUAAA, AUUAAA, and variants of AAUAAA are generally considered as canonical hexamers and account for 88% of poly(A) sites in human genes (Tian and Graber, 2012). In order to further elucidate the poly(A) signals in *X. tropicalis*, the top 50 hexamers in the PE region were obtained. Previous studies have demonstrated that the patterns with spatial preference near the poly(A) site are involved in specific interaction with the polyadenylation mechanism (Beaudoing et al., 2000); accordingly, we identified patterns that are distributed concentratedly (**Figure 3D**). We used the variance to quantitatively represent the extent of spatial aggregation of each pattern. The real frequency and the expected frequency of each *k*-gram in PE were calculated, and the corresponding *p*-value based on *t*-test was obtained to further identify over-representative poly(A) signals. Finally, considering the standard deviation around the mean position of a hexamer, we found 12 hexamers (*p*-value < 0.00001), which are significantly over-represented near the poly(A) site (**Table 2**). These 12 signals are spatially distributed, which is similar to known poly(A) signals in humans (Beaudoing et al., 2000). Compared with the significant poly(A) signals in humans (AAUAAA, AUUAAA, AGUAAA, UAUAAA, CAUAAA, GAUAAA, AAUAUA, AAUACA, AAUAGA, AAAAAG, and ACUAAA) (Beaudoing et al., 2000), the two most prominent signals in *X. tropicalis* are as in animals, which are AAUAAA and AUUAAA. The 12 over-represented signals in *X. tropicalis* are further divided into two categories. One category contains significant signals in both humans and *X. tropicalis*, including AAUAAA, AUUAAA, UAUAAA, AGUAAA, AAUACA, CAUAAA, GAUAAA, AAUAUA, and ACUAAA. The other category consists of signals that are only significant in *X. tropicalis*, including AUAUAU, AAAAAA, and AAUGAA. The unique signals in *X. tropicalis* may be due to the difference in GC content between the two species because of the more significant DSE element upstream of poly(A) sites in *X. tropicalis*.

**Table 2:** Significant patterns in 3′ UTR poly(A) sites.

Hexamer	Observed^1^	Expected^2^	Coverage^3^	Position^4^	P^5^	Location^6^
**AAUAAA**	6,609	1,303	47.7%	−23 ± 4.1	0	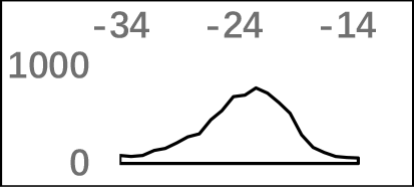
**AUUAAA**	2,347	419	26.2%	−23 ± 4.3	0	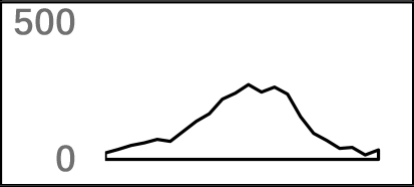
**UAUAAA**	852	226	21.5%	−24 ± 4.9	0	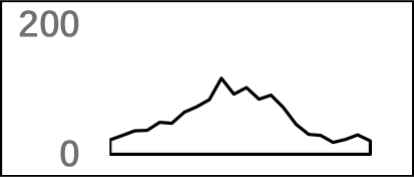
**AGUAAA**	485	112	11.3%	−23 ± 4.6	2 × 10^−280^	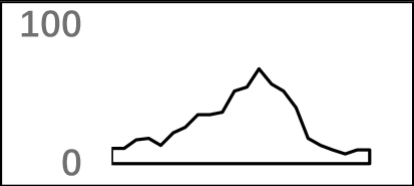
**AUAUAU**	465	96	20.1%	−23 ± 6.2	1 × 10^−277^	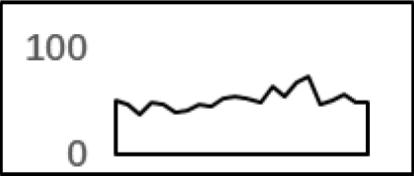
**AAUACA**	393	81	13.5%	−23 ± 5.1	2 × 10^−270^	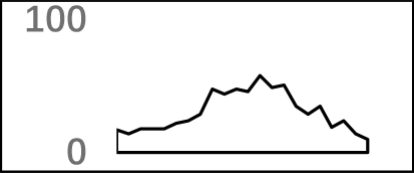
**AAAAAA**	1,111	359	19.5%	−23 ± 6.0	6 × 10^−181^	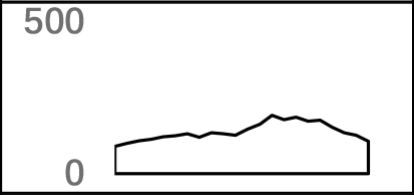
**CAUAAA**	342	77	10.8%	−24 ± 5.0	3 × 10^−205^	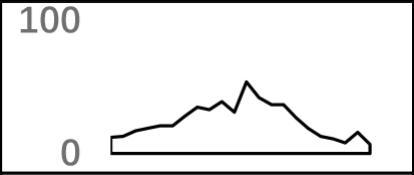
**GAUAAA**	228	57	8.0%	−24 ± 4.8	3 × 10^−113^	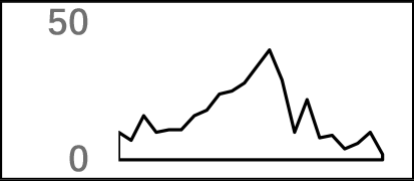
**AAUAUA**	192	67	19.9%	−23 ± 5.4	1 × 10^−53^	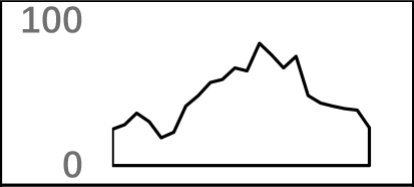
**AAUGAA**	200	73	15.2%	−23 ± 6.0	8 × 10^−51^	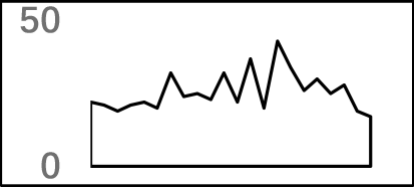
**ACUAAA**	151	51	8.6%	−24 ± 5.8	2 × 10^−45^	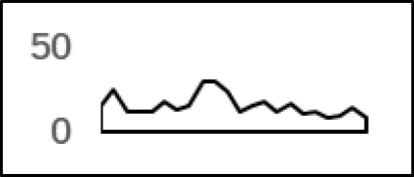

^1^The occurrence of hexamer in PE region of all poly(A) sites.

^2^The expected occurrences based on the first-order Markov chain model in PE region of all poly(A) sites.

^3^Coverage of hexamers in the PE region of all poly(A) sites.

^4^A position is denoted by average ± SD. The mean position of each pattern in the PE region and the standard deviation (SD) were used as a measure of scattering.

^5^The probability of reaching the observed frequency by chance based on a cumulative binomial distribution.

^6^Distribution of hexamers in the PE region.

### Schema of Polyadenylation Signals of Proximal APA Sites

**Figure 4:**
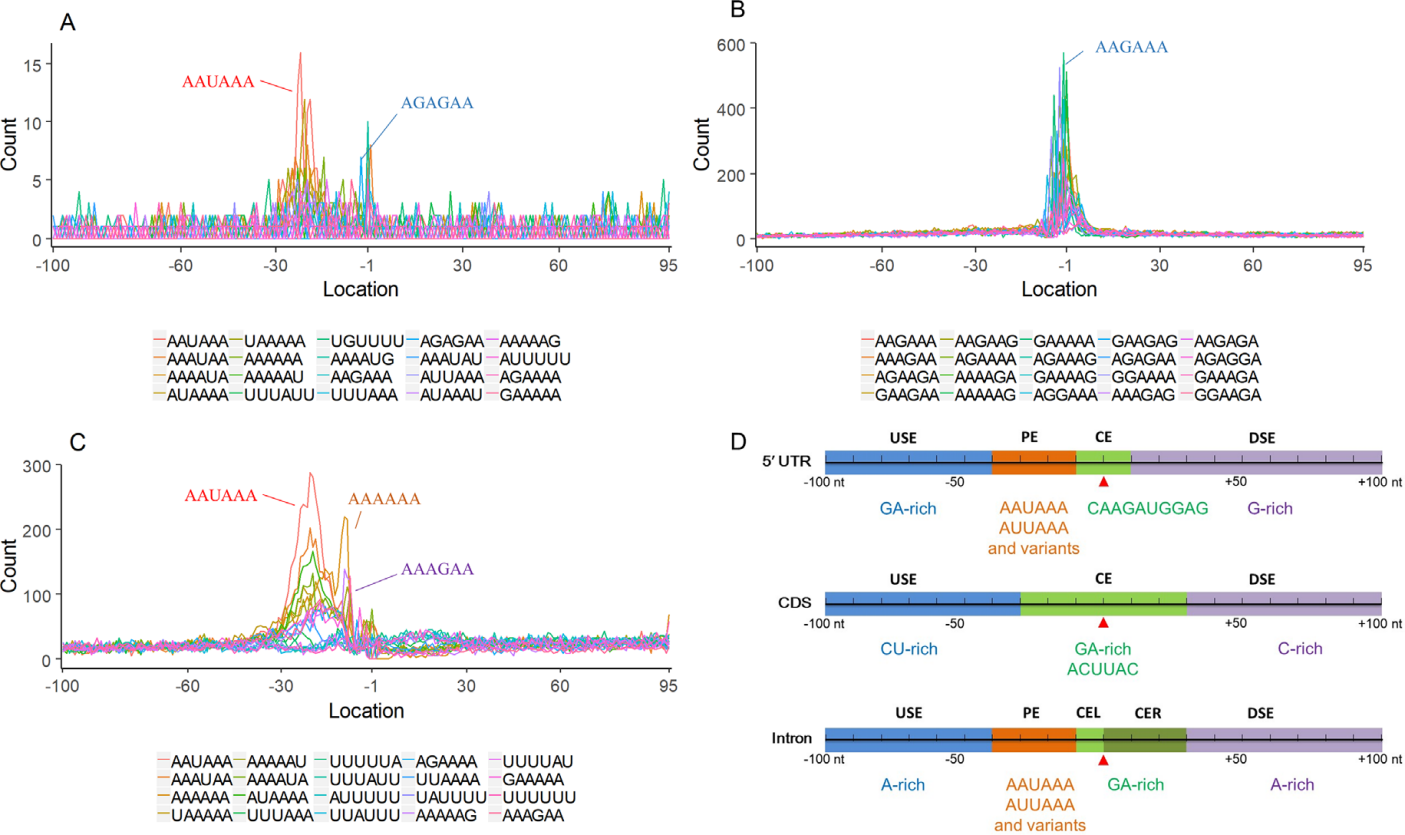
Signal distribution of non-3′ UTR APA sites in *X. tropicalis*. **(A)** Top 20 hexamers according to the occurrence number from −100 to 100 bp around 5′ UTR poly(A) sites. The signal has changed dramatically in the −40 nt to −1 nt. The most significant signal in PE region is AAUAAA, similar to the PE signal on the 3′ UTR. **(B)** Top 20 hexamers according to the occurrence number from −100 to 100 bp around CDS poly(A) sites. The most significant hexamer is ACUUAC, and the highly overlapping hexamer is AAGAAA in CE. **(C)** Top 20 hexamers sorted according to the occurrence number from −100 to 100 bp around intronic poly(A) sites. The most significant signal is AAUAAA in PE, and there is a GA-rich element around CS. **(D)** Polyadenylation signal models on 5′ UTR, CDS, and intron. The red triangle denotes the poly(A) site. USE, upstream sequence element; PE, positioning element; CE, cleavage element; CEL, left cleavage element; CS, cleavage site; CER, right cleavage element; DSE, downstream sequence element.

Nearly 50% of poly(A) sites from ∼68% genes were found in CDS, intron, and 5′ UTR regions. Distinct characteristics of nucleotide compositions from 3′ UTR poly(A) sites were found in these unconventional sites (**Figures 4** and **S4** vs. **Figures 2A** and **S3**). To further investigate signals of poly(A) sites located in different genomic regions, hexamer distributions and over-represented hexamers were examined (see Materials and Methods).

Compared with the 3′ UTR profile (**Figure 2A**), A content of the 5′ UTR poly(A) site is generally higher. There is no sudden increase of U content downstream of the cleavage site, and the difference between the G and C contents is more dramatic (**Figure S4A**). By searching for the top 20 hexamers, we found that the signal changes were concentrated between −30 nt and −10 nt (**Figure 4A**), and the signal exhibited two peaks. We combined the single-nucleotide distribution with the hexamer distribution and referred to the 3′ UTR signal model (**Figure 2D**) to divide the entire signal region into four parts: USE (−100 nt ∼ −40 nt), PE (−40 nt ∼ −10 nt), CE (−10 nt ∼ 10 nt), and DSE (10 nt ∼ 100 nt) (**Figure 4D**). We analyzed the frequency of occurrence of the hexamer on each signal region based on the oligonucleotide analysis function (see Materials and Methods). The most significant hexamer in the USE region is GAGAGA. All of the selected hexamers can be assembled into multiple consensus sequences (**Table S8**). The best positional element on USE can be abbreviated as “GA-rich.” The most significant hexamers on PE are AAUAAA, AUUAAA, and their variants, which are very similar to the 3′ UTR signal. The most significant hexamer on CE is AGAUGG. The consensus sequence on CE is CAAGAUGGAG. The most significant hexamer on the DSE region is GAGAGA, and the consensus sequence is GAGGAGAGACA, which can be abbreviated as “DSE.”

In contrast, the nucleotide distribution of CDS poly(A) sites is completely different from that of 3′ UTR sites. The peak of A content no longer appears on the upstream of the site, and the U content continues to decrease until it is less than the G content at the vicinity of CS (**Figure S4B**). The poly(A) signal of CDS sites is highly concentrated near the cleavage site (−12 nt to 12 nt) and is a more DSE component with the dominant pattern AAGAAA (**Figure 4B**). We divide the entire signal region into three parts: USE (−100 nt ∼ −30 nt), CE (−30 nt ∼ 30 nt), and DSE (30 nt ∼ 100 nt) (**Figure 4D**). The top two significant hexamers on USE are UCAUCA and CUCCUC (**Table S8**). The consensus sequence on USE is AUCAUCU, which is abbreviated as “CU-rich.” The most significant hexamer is ACUUAC on CE, and the highly overlapping hexamer is AAGAAA. The best consensus sequence is ACUUACCUUUU, which includes the most over-represented hexamer, ACUUAC. The other set of hexamers forms a completely different motif, the consensus sequence of which is AAGAAGAAA, which includes the highly overlapping hexamer, AAGAAA. These consensus sequences can be simply described as “GA-rich.” The most significant hexamer on DSE is CUCCUC, and the consensus sequence is CCUCCUCC, which can be abbreviated as “C-rich.” Generally, the most important poly(A) signal element for CDS sites is CE, which is GA-rich.

We found that ∼50% genes have at least one poly(A) site in intron (**Table S3**). Particularly, almost half of genes have multiple poly(A) sites in intron. Although nucleotide distribution of intronic poly(A) sites is generally similar to that of 3′ UTR sites, the peak of A content was not observed around intronic poly(A) sites (**Figure S4C**). We divide the entire signal region into five parts: USE (−100 nt ∼ −40 nt), PE (−40 nt ∼ −10 nt), CEL (−10 nt ∼ −1 nt), CER (1 nt ∼ 30 nt), and DSE (30 nt ∼ 100 nt) (**Figure 4D**). The top three significant hexamers on USE are AAAAAA, AAUAAA, and GAAGAA (**Table S8**). The corresponding consensus sequence on USE is UAAAUAAA. The most important and highly overlapping hexamer on PE is AAUAAA, which is the same as for 3′ UTR sites. The top three significant hexamers on CEL are CAGUAG, AGUAGG, and CCCUAC, and these hexamers constitute a DSE consensus sequence of ACAGUAGGGCAA. The top three significant hexamers on CER are GGAGAC, GAUGGA, and GAGACA, and the consensus sequence is “GA-rich.” On DSE, AAUAAA, CCUUUA, and GAGAGA are the most over-represented, and the corresponding consensus sequence is AUAAAUAAA.

### APA Dynamics Across Developmental Stages in *X. tropicalis*

**Figure 5:**
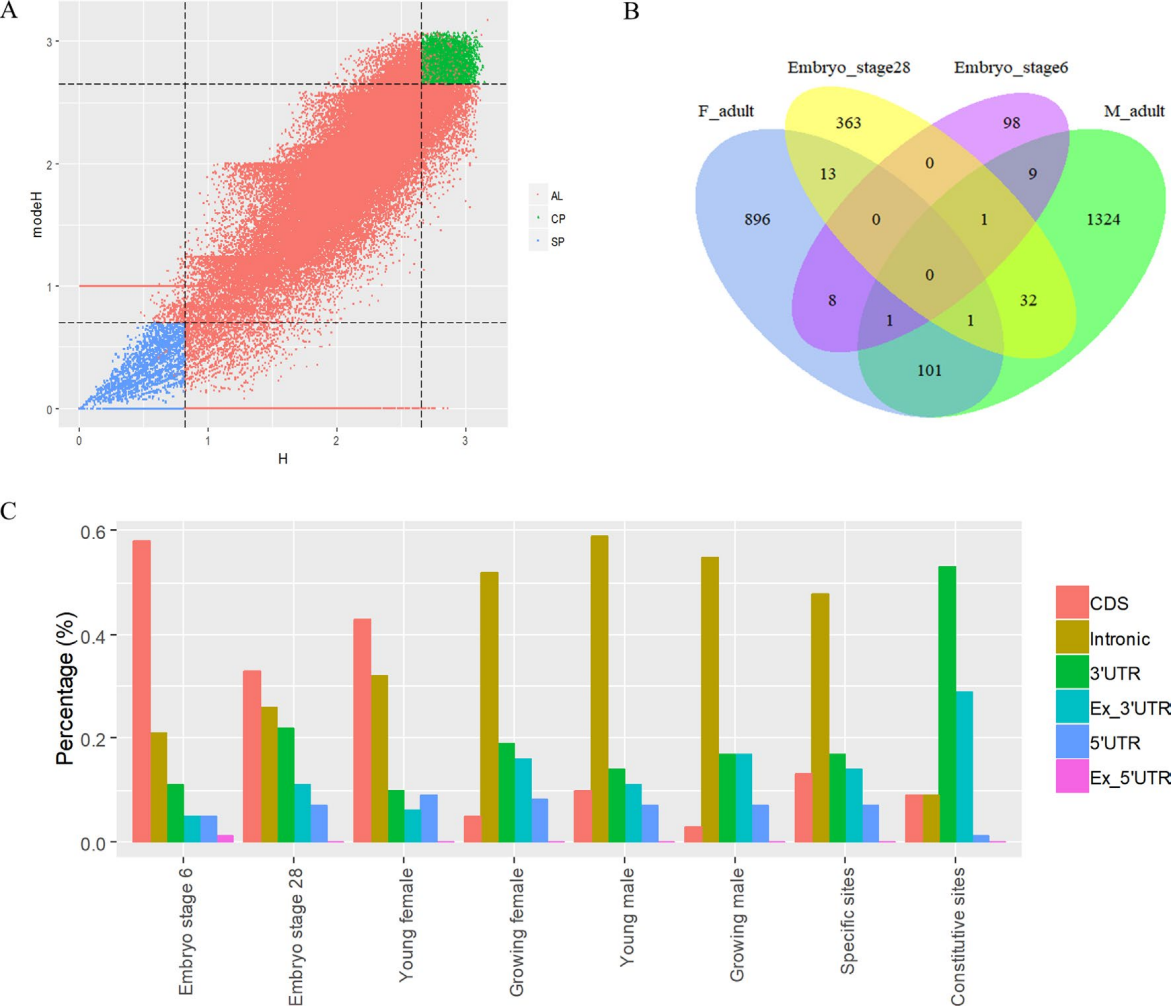
Characteristics of poly(A) signals during the development of *X. tropicalis*. **(A)** Information entropy distribution of poly(A) sites. The *x*-axis is the conventional information entropy (H), and the dashed lines represent thresholds of 0.82 and 2.66. The *y*-axis represents the adjusted information entropy (modeH), and the dashed lines represent thresholds of 2.65 and 2.70. Each point denotes one poly(A) site. Specific site is colored in blue, and constitutive site is colored in red. Red points in the top-right and bottom-left corners are sites that were selected based on the entropy value but were not defined as specific or constitutive sites because of their low expression levels (supported by less than five reads). AL: all poly(A) sites; SP: specific sites; CP: constitutive sites. **(B)** Venn diagram showing the overlap of specifically expressed genes at different developmental stages. “F_adult” includes young females and growing females. “M_adult” includes young males and growing males. **(C)** Percentages of specific poly(A) sites located in different locations across different periods. We randomly selected the same number of specific sites and constitutive sites and calculated the percentage of the genomic regions where these sites are located. In this figure, we only selected six periods (embryo stage 6, embryo stage 28, young female, growing female, young male, and growing male) with sufficient quantity for analysis.

To analyze the dynamics of APA site usage across developmental stages in *X. tropicalis*, we first distinguished developmental stage-specific poly(A) sites that are uniquely expressed in one stage from constitutive sites that are expressed in more than one stage. According to the sample information of the raw data (Zhou et al., 2019), the entire life span stages are divided into nine stages, including the embryonic stages 6 and 8 before the maternal-to-zygotic transition (MZT); embryonic stages 11, 15, and 28 after the MZT; and four adult stages that includs young males, growing males, young females, and growing females. We used an outlier detection method called ROKU to calculate the general information entropy (H) and the improved information entropy (modeH) for each poly(A) site (Kadota et al., 2006). Both H and modeH range from 0 to 4. The lower the entropy value of a poly(A) site is, the higher the specificity is. Poly(A) sites were ranked by H and modeH values; then the bottom 5% sites (3,500 sites with H > 2.66 and modeH > 2.65) were defined as specific, and the top 5% sites (3,500 sites with H < 0.82 and modeH < 0.70) were defined as constitutive (**Figure 5A**). We also defined period expressed sites as the sites that are expressed at a given stage regardless of their expression level in other stages. The base compositions surrounding specific poly(A) sites and constitutive ones are generally similar, while specific sites are with lower A content in the PE region and lower U content in CER (**Figure S5**). Next, the distribution of top 20 hexamers around specific sites and constitutive sites were explored, and results showed that poly(A) signals at specific sites have more dramatic fluctuations on USE while weaker fluctuations on CER (**Figure S6**).

The APA frequency of specific sites varies across different developmental stages. Among the 3,500 developmental stage-specific poly(A) sites, sites specific to each developmental stage were identified based on their expression levels across different stages. Among the nine stages, embryo stage 6, embryo stage 28, and the four adult stages contain more APA sites and highly expressed genes than do other stages (**Figure S7**), which suggests that these stages may be more involved in growth than other embryonic stages. Interestingly, embryo stage 6 and 28, adult female stage, and adult male stage share few annotated genes that contain specific sites (**Figure 5B**), and most of the stage-specific genes are exclusively expressed in their respective period. By comparing the APA ratio of specific APA sites and the ratio of period expressed sites over these periods (**Figure S8A**), we found that the overall APA ratios of period expressed sites are three times those of specific APA sites (average value: 3.30 vs. 1.08), which means that very few stage-specific sites map to the same gene. In addition, the APA ratio for both types of sites remained stable before MZT (embryo stages 6 and 8), and it was abrupt after MZT (embryo stages 11, 15, and 28). This is because the APA site regulates developmental events after MZT due to zygotic genome activation (Zhou et al., 2019).

The APA frequency of specific sites and constitutive sites is also different between females and males (**Figure S8B and C**). In adult individuals, females and males perform extremely different at the frequency of the two types of APA sites. In females, the number of constitutive sites remains essentially constant, and the number of period expressed sites decreases from the young to the growing stage (**Figure S8D**), but the number of specific sites increases dramatically. However, there is an opposite trend in males. From the young to growing stage, the number of constitutive sites and the number of specific sites increased to some extent. At the same time, we found that even though the APA frequency varies greatly between genders, the number of APA genes is not significantly changed. Females have 14,985 APA genes during the young stage and 15,009 genes in the growing stage, while the number of genes in males during these two periods was 14,216 and 15,080, respectively. This result depicts the dynamics of APA site usage rather than the gene usage related to gender in the development of each individual.

The APA frequency of specific sites in different genomic regions varies with developmental stages (**Figure 5C**). We use the 3,500 specific sites and 3,500 constitutive sites to analyze their distributions in different genomic regions. With the use of the constitutive sites as the control, it was found that specific sites are mainly distributed in proximal regions (**Figure 5C**). However, more than 82% of constitutive sites are located in 3′ UTR or 3′ UTR extended regions. As the development progresses, the proportion of sites located in CDS gradually decreases, and the proportion of sites located in intron gradually increases. During the embryonic period, most of the specific sites are concentrated on the intron, but in the late development, most of the specific sites are concentrated on the CDS. These sites located in intron or CDS regions may contribute to the dynamics of gene expression during developmental stages.

## Discussion

In this study, we conducted a systematic investigation of genome-wide poly(A) sites in *X. tropicalis*. By analyzing gene regulatory elements at the single base level, we found five major *cis* elements (USE, PE, CEL, CER, and DSE; **Figure 2D**) and 12 patterns that are significantly over-represented in 3′ UTR APA sites of *X. tropicalis* (**Table 2**). These patterns not only play different roles but also continue polyadenylation in the event of failure or absence of certain signals, ensuring a high degree of flexibility for polyadenylation. Among them, several patterns have been proved experimentally to be functional in the polyadenylation of *Xenopus*. For example, AAUACA, a variant of AAUAAA, was confirmed to ensure the polyadenylation when AAUAAA failed in *Xenopus laevis* (Mason et al., 1985). CAUAAA was reported to be effectively used in *X. laevis* α-tubulin gene XαT14 (Rabbitts and Morgan, 1992). The U-rich region upstream of *X. tropicalis* and AAUAAA jointly promote the 3′ UTR lengthening of cytoplasmic mRNAs (Sheets et al., 1994). AAGAAA, also an AAUAAA mutant, may be associated with clam p82, a functional homolog of *Xenopus* CPEB, and plays an active role in polyadenylation (Minshall et al., 1999). The 12 patterns identified in this study cover 92% of 3′ UTR APA sites in *X. tropicalis*, which is comparable with 88% in humans (Beaudoing et al., 2000). However, of all poly(A) sites located in protein-coding genes, the proportion of 3′ UTR APA in *X. tropicalis* genes is 30.15% (**Table S3**), which is much lower than the 54% in humans (Tian et al., 2005). This may be partially due to the incomplete 3′ UTR annotations or inaccurate transcript structures in current genome annotation (NCBI v9.1) of *X. tropicalis*. The compendium of poly(A) sites provided in this study would provide valuable resources for improving the genome annotation in *X. tropicalis*.

In addition to 3′ UTR poly(A) sites, a large number of sites were found in proximal regions, including 5′ UTR, CDS, and intron (**Table S2**), which is consistent with the observation in previous studies that an increasing number of noncanonical APA sites were detected (Wu et al., 2011; Sherstnev et al., 2012; Guo et al., 2016; Chen et al., 2017). To understand how these noncanonical APA sites were recognized, we comprehensively explored their poly(A) signals and proposed distinct schemas of *cis* elements for different groups of poly(A) sites (**Figure 4D**). Poly(A) sites from different groups were flanked by regions with distinct nucleotide composition preferences (**Figure S4**). The most intuitive difference between the 3′ UTR and non-3′ UTR polyadenylation signals is the higher expression of G-rich elements surrounding non-3′ UTR sites (**Figure 4D**). Particularly, the profile of CDS sites is very different from that of sites from the other three genic locations (**Figure S4**). For CDS poly(A) sites, AAUAAA and its variants were not observed, but several signals such as AAGAAA, AAGAAG, and AAGAGA accumulated around the cleavage site, which may signify the presence of new polyadenylation factors or other unknown proteins to recognize these signals (**Figure 4D** and **Table S18**). Similar nucleotide compositions of CDS poly(A) sites were also found in plant (Tian et al., 2007; Wu et al., 2011; Sherstnev et al., 2012; Guo et al., 2016). Polyadenylation in introns can lead to the conversion of an internal exon to a 3′ terminal exon or usage of a 3′ terminal exon that is otherwise skipped. Although some intronic APA sites may be derived from transcriptional read-through or gene mis-annotations (Wu et al., 2015), the previous study has demonstrated that the dynamic interplay between polyadenylation and splicing leads to extensive polyadenylation in introns and contributes to the complexity of transcriptome in the cells (Tian et al., 2007). In this study, we found that the gene length and intron numbers have an impact on APA frequency, and they are highly correlated with the frequency of proximal rather than 3′ UTR APA sites (**Figure 1D**). It seems that the longer sequences may enhance the likelihood of APA emergence. To clarify the cause of this effect, here, we calculated the average length of introns and then calculated the correlation coefficient among the APA frequency, the gene length, the number of introns, and the average length of the introns (**Figure S9**). Interestingly, although the length of the gene has a prominent influence on the average length of the intron (*r* = 0.56, *p*-value < 2.2 × 10^−16^), the average length of the intron is not much related to the frequency of proximal APA usage (*r* = 0.22, *p*-value < 2.2 × 10^−16^). On the contrary, the number of introns has a greater influence on the frequency of the proximal APA sites (*r* = 0.41, *p*-value < 2.2 × 10^−16^). This demonstrates that proximal APA frequencies are positively correlated with their genetic signatures, including the number of introns and gene length, and essentially raise the frequency of proximal APA sites by increasing the number of introns. A much smaller number of APA sites were found in 5′ UTR (5%) (**Table S2**), compared with CDS or intron site. Both the cap structure at the 5′ UTR and the poly(A) tail at the 3′ UTR are acting synergistically as important factors for translation (Chizhikov and Patton, 2000). A recent study on HIV-1 genome revealed that a functional poly(A) signal is required for viral packaging and AAUAAA within the 5′ polyadenylation domain is a dual regulator of gRNA production and packaging (Smyth et al., 2018). Our study also found AAUAAA as the most significant pattern in PE region of 5′ UTR poly(A) sites (**Table S8**). In addition, we found several other G-rich or GA-rich elements around 5′ UTR sites, which may be considered as auxiliary signals for 5′ polyadenylation. Collectively, these results shed light on the understanding of noncanonical APA and provide insights into the underlying mechanisms for non-3′ UTR polyadenylation and its regulation in *X. tropicalis*.

Understanding of molecular mechanisms involved in the regulation of embryonic development represents one of the hot topics in developmental biology. Our study aims to investigate the dynamic changes in APA characteristics during embryonic development. In the present study, we found that the numbers of APA sites and their associated genes vary from embryos to adults along different stages during a life span (**Figure S8**). Overall, the number of genes remained relatively consistent, while the number of APA sites varied dramatically, especially between different genders. This phenomenon further proves the previous conclusion (Zhou et al., 2019) that APA usage rather than gene usage depends on gender. Then, poly(A) sites specific to the developmental stage are further identified by the principle of information entropy. Since these specific sites are actually the most prominent sites at their respective stages, the sites between the various stages do not overlap, but the genes of these sites are overlapped at different developmental stages. In this study, we did find genes that overlap over several periods (**Figure 5B**), even though the number of these genes is small. It is undeniable that less overlap may be due to the insufficient sample of specific sites in each period. Interestingly, there are few overlapping genes in the pre-embryo and late embryo, but there is an increase in the overlap between the late embryo and the adult, and there is a difference in the number between male and female individuals. These differential overlapping genes may be related to gender, which requires subsequent genetic functional analyses to be further confirmed. Using constitutive sites as a control group, we analyzed the location of the randomly selected equal number of poly(A) sites on the gene. The results show that a large number of specific sites are located in proximal regions, especially in introns, because zygote-activated transcripts are the shortest. These proximal APA sites may affect gene-specific expression, while genes that undergo polyadenylation at 3′ ends are more likely to maintain basic cellular function and are expressed throughout the development processes. Our results provide evidence for a major regulatory role for APA on the proximal region of tissue-specific genes. Further research is needed to investigate the molecular mechanisms of APA regulation in proximal during embryonic development.

Taken together, our study comprehensively analyzed poly(A) site signals of 3′ UTR and proximal poly(A) sites in *X. tropicalis*. We constructed distinct poly(A) signal models to describe potential *cis* elements of 3′ UTR and non-3′ UTR poly(A) sites in *X. tropicalis*. We found 12 significant patterns of conventional 3′ UTR sites with three exclusively present in *X. tropicalis*. For proximal poly(A) sites, we divided them into three categories according to their genomic locations and compared the difference of signal characteristics among different categories of sites. Distinct poly(A) signals were found for APA sites in 5′ UTR, CDS, and intron. Our results on poly(A) signal variation among different groups of APA sites could be incorporated in poly(A) site or gene prediction programs for better performance. We also showed that the effect of genetic characteristics (the number of introns, gene length, and intron length) of proximal sites on APA frequency is actually caused by increasing the number of introns. Finally, we analyzed dynamic APA usages across different developmental stages. This work reports a comprehensive study of the poly(A) signal characteristics between the distal and proximal poly(A) sites in *X. tropicalis* and provides new insights into the regulation of APA during development.

## Data Availability Statement

Publicly available datasets were analyzed in this study. This data can be found here: https://www.ncbi.nlm.nih.gov/geo/query/acc.cgi?acc=GSE74919.

## Author Contributions

GJ conceived the study; GJ, ZJ, and XW managed and coordinated the project; ZJ provided data sources; SZ performed the experiments and analyzed the data; CY, MC, and HF contributed to the discussion of the content; SZ, XW, and ZJ wrote the paper. All authors participated in manuscript writing and approved the final version of the manuscript.

## Funding

This work was supported by the National Natural Science Foundation of China (No. 61573296 to GJ, No. 61871463 to XW, and No. 61802323 to CY), Natural Science Foundation of Fujian Province of China (No. 2017J01068 to XW), and the Fundamental Research Funds for the Central Universities in China (Xiamen University: 20720170076 to CY). This work was also supported by the Eunice Kennedy Shriver National Institute of Child Health & Human Development of the National Institutes of Health under Award Number R21HD076845 and the National Institute of Food and Agriculture, United States Department of Agriculture, under Award Number 2016-67015-24470 to ZJ.

## Conflict of Interest Statement

The authors declare that the research was conducted in the absence of any commercial or financial relationships that could be construed as a potential conflict of interest.
