# Supplementary material for: Modeling of Genome-Wide Polyadenylation Signals in Xenopus tropicalis
Source: Front Genet. 2019 Jul 3;10:647. doi: 10.3389/fgene.2019.00647 (PMC6616101; doi:10.3389/fgene.2019.00647)
Supplement: Supplementary file 1 [file DataSheet_1.pdf]

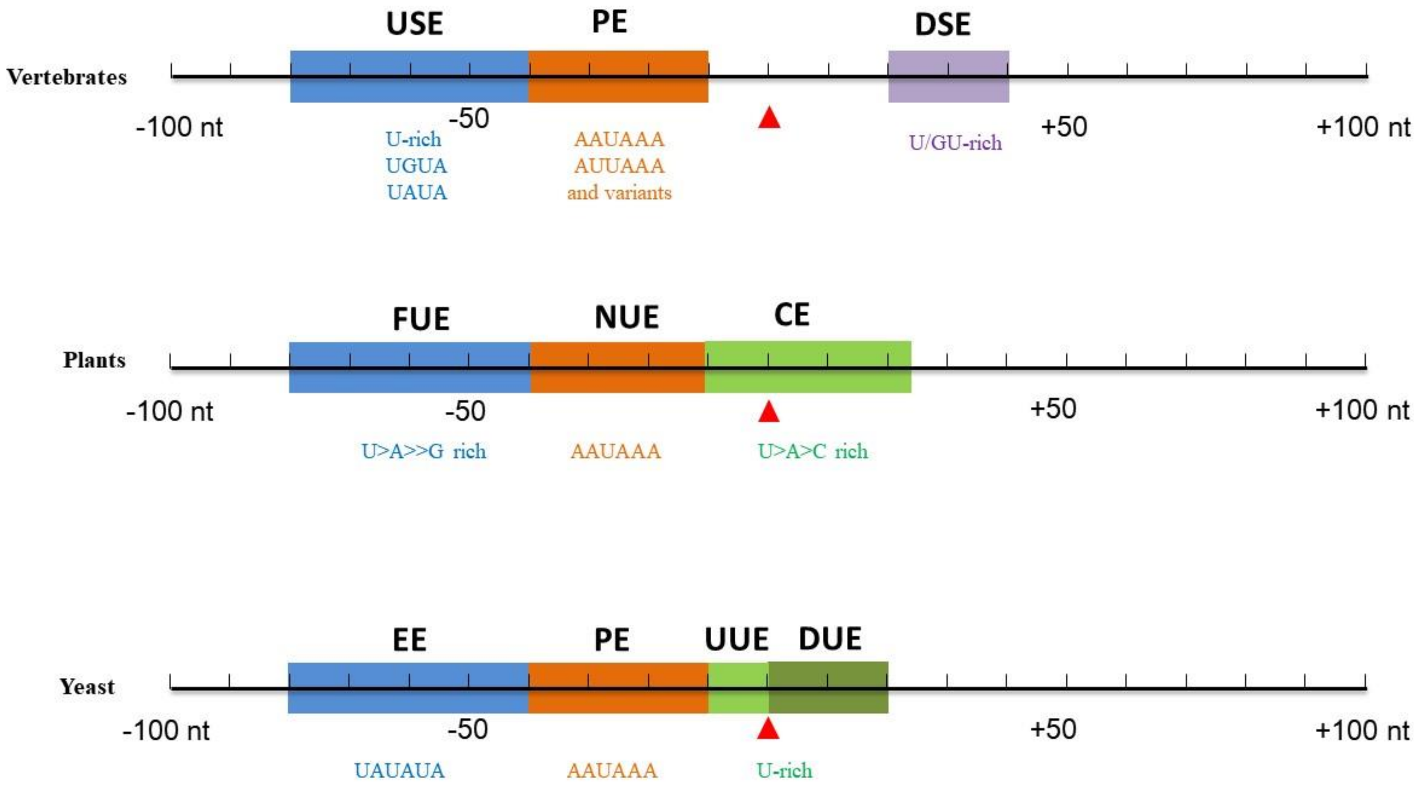

Figure S1. Poly(A) signal models of different species. The red triangle denotes the poly(A) site. USE, upstream sequence element; PE, positioning element; CE, cleavage element; DSE, downstream sequence element; FUE, far upstream element; NUE, near upstream element; EE, efficiency element; UUE, upstream U-rich element; DUE, downstream U-rich element.

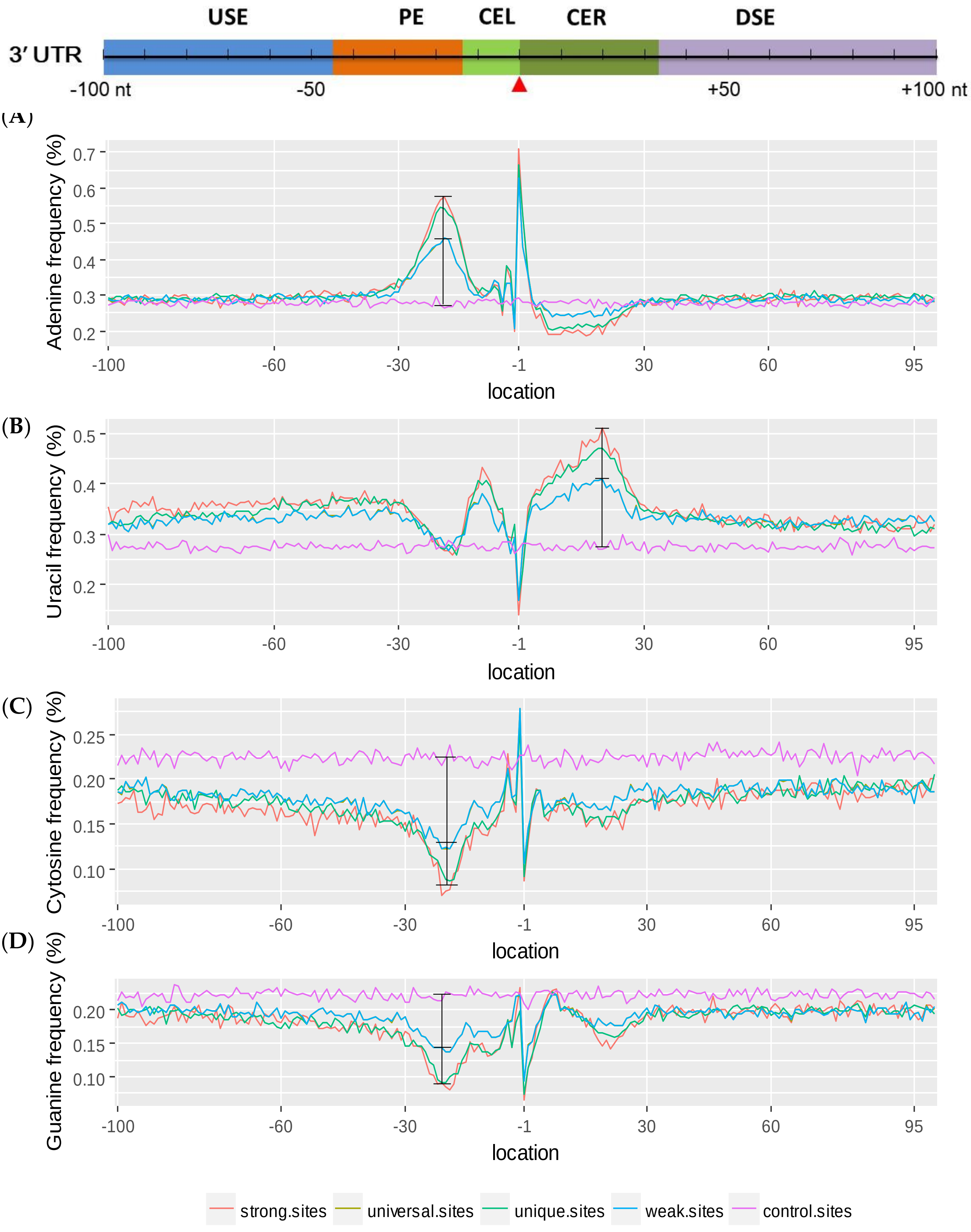

Figure S2. Distribution frequency of the four bases on the 200 nt sequence around the five type of 3′ UTR sites. (A) Distribution of adenine in five types of poly(A) sites; (B) Distribution of uracil in five types of poly(A) sites; (C) Distribution of cytosine in five types of poly(A) sites; (D) Distribution of guanine in five types of poly(A) sites. The schema of poly(A) signal regions is displayed on the top.

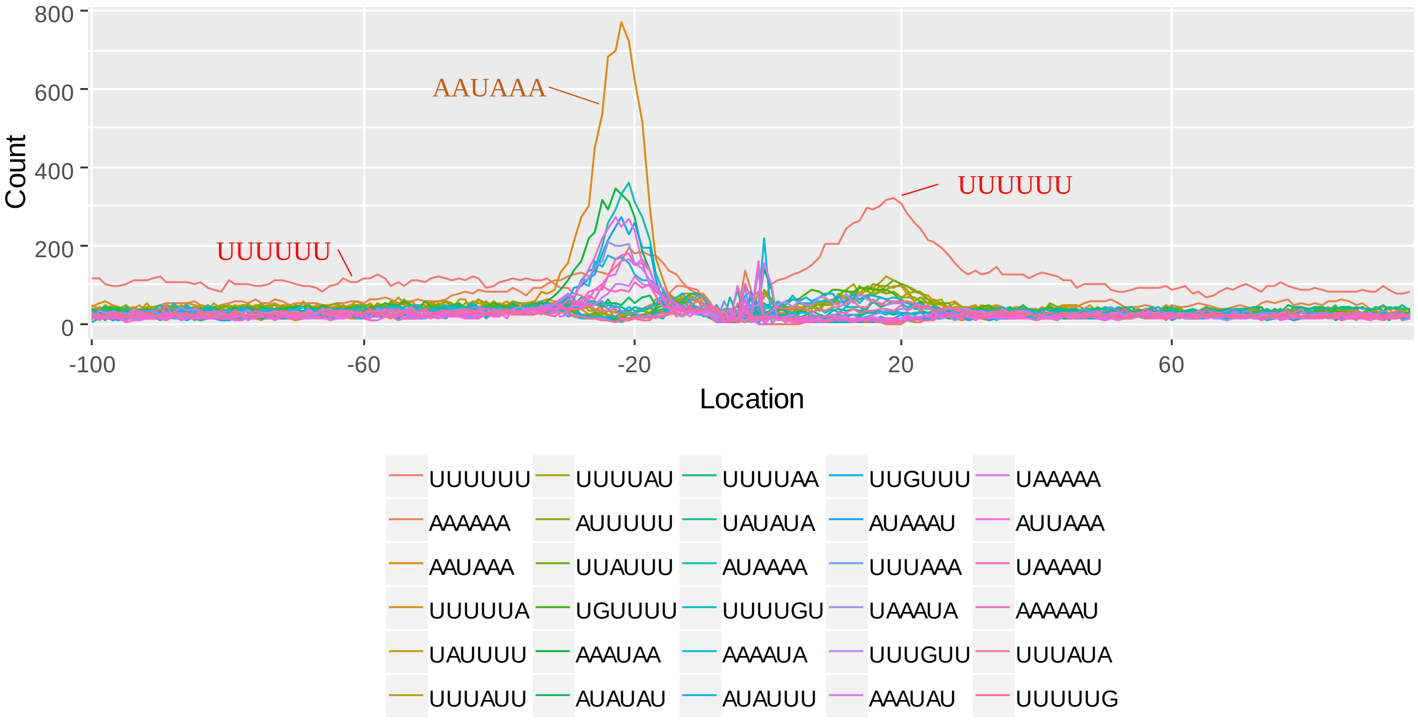

Figure S3. Top 30 hexamers around 3′ UTR poly(A) sites.
Only the top 30 hexamers are shown.

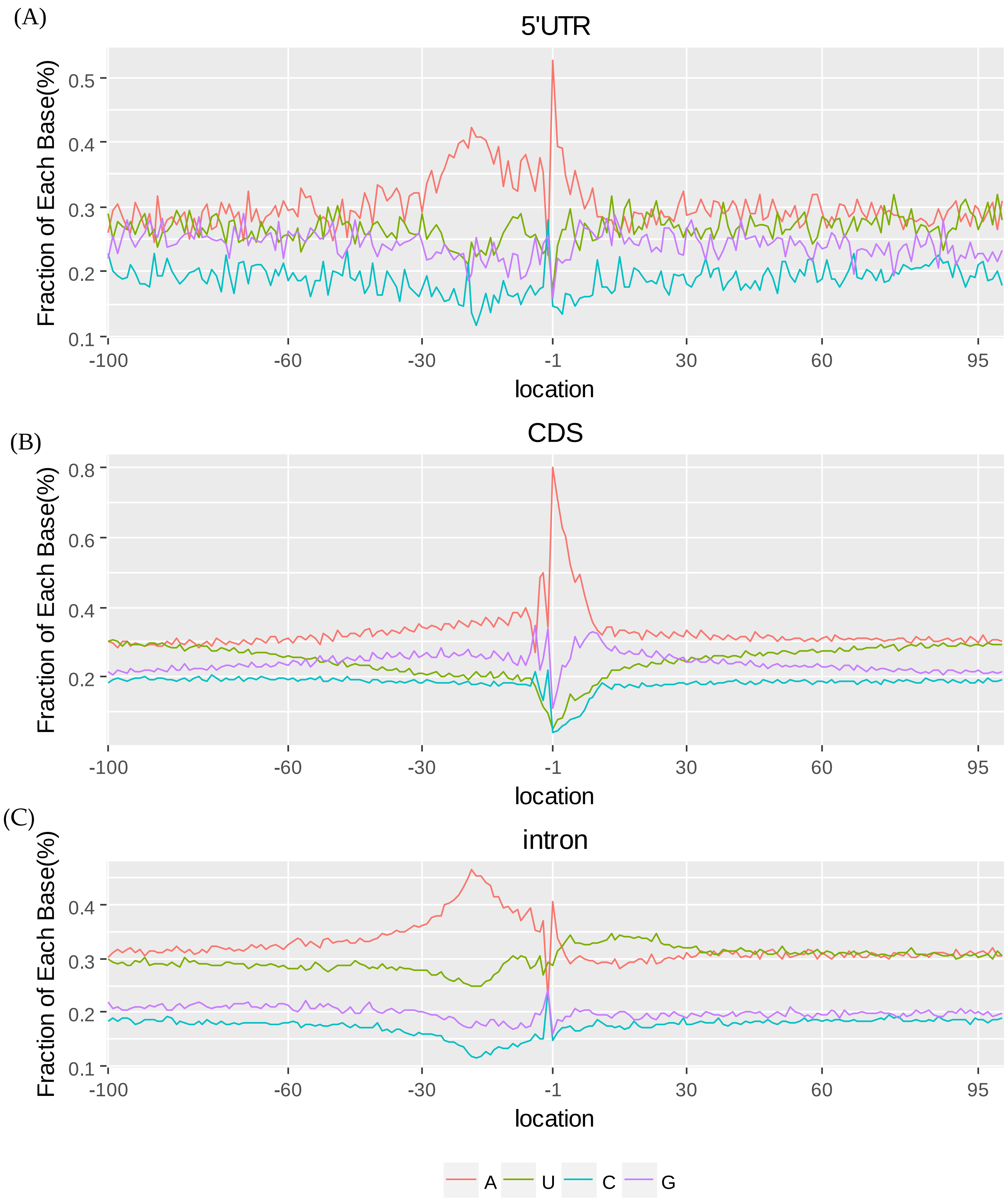

Figure S4. Single nucleotide profile surrounding poly(A) sites in non-3′ UTR regions of 5′ UTR (A), CDS (B), intron CDS (C).

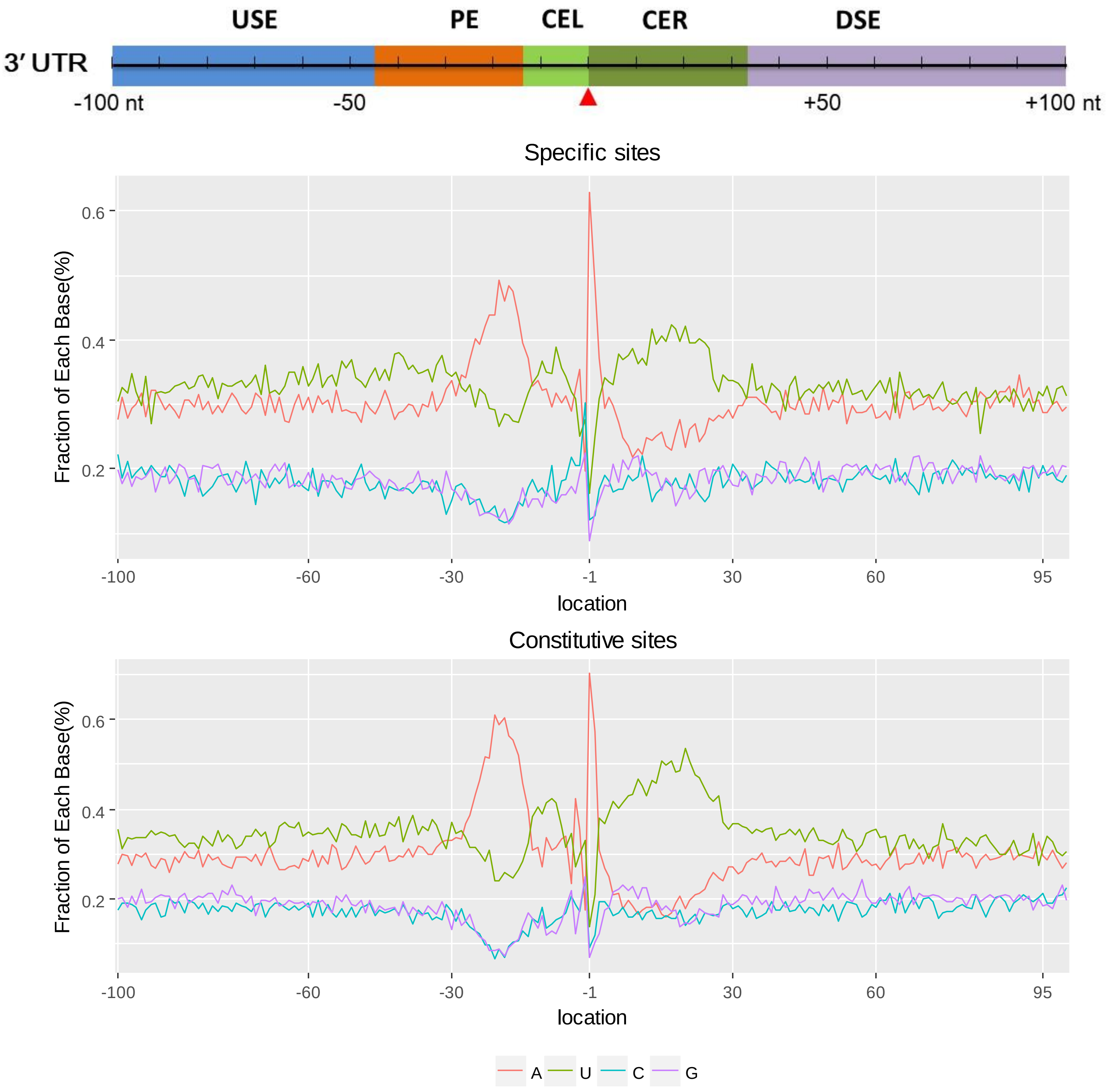

Figure S5. Single nucleotide profiles of specific poly(A) sites and on constitutive sites. The schema of poly(A) signal regions is displayed on the top.

(A)

(B)

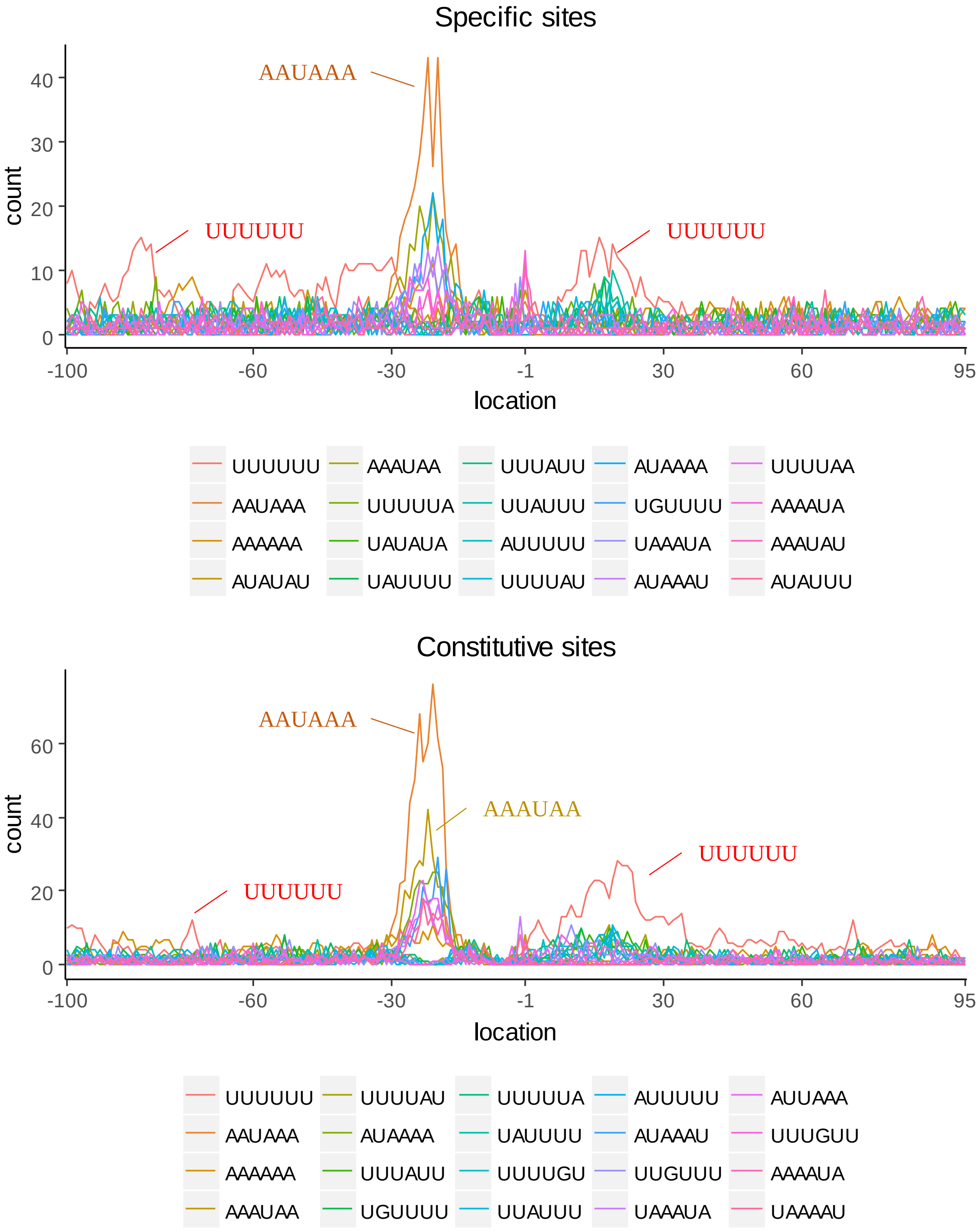

Figure S6. Distribution of hexamers around specific sites and constitutive sites.

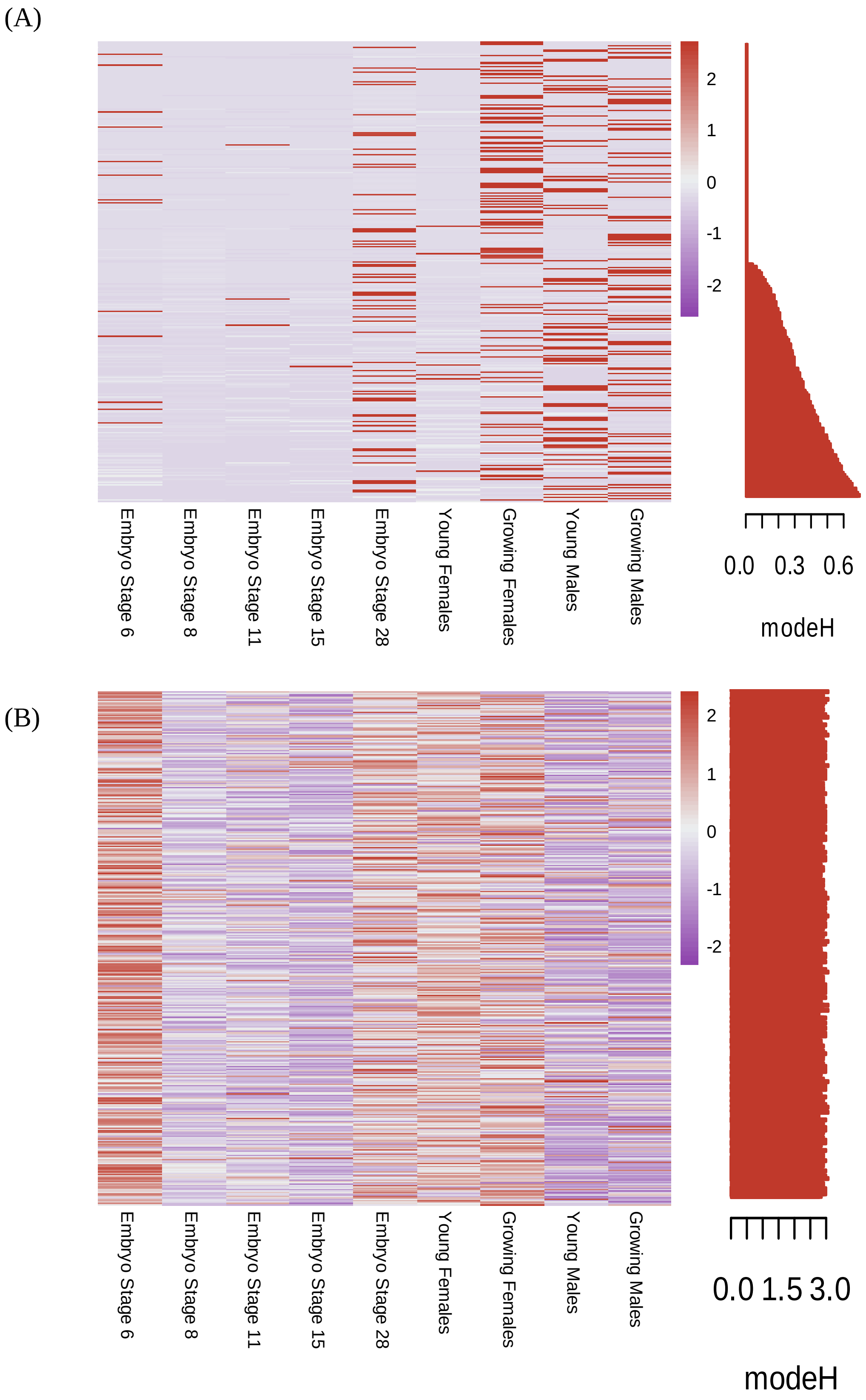

Figure S7. Expression pattern of APA sites. (A) Expression pattern of specific sites; (B) Expression pattern of constitutive sites. The right column shows the value of the adjusted information entropy corresponding to the left heat map. Rows of the heat map are ordered by the entropy value.

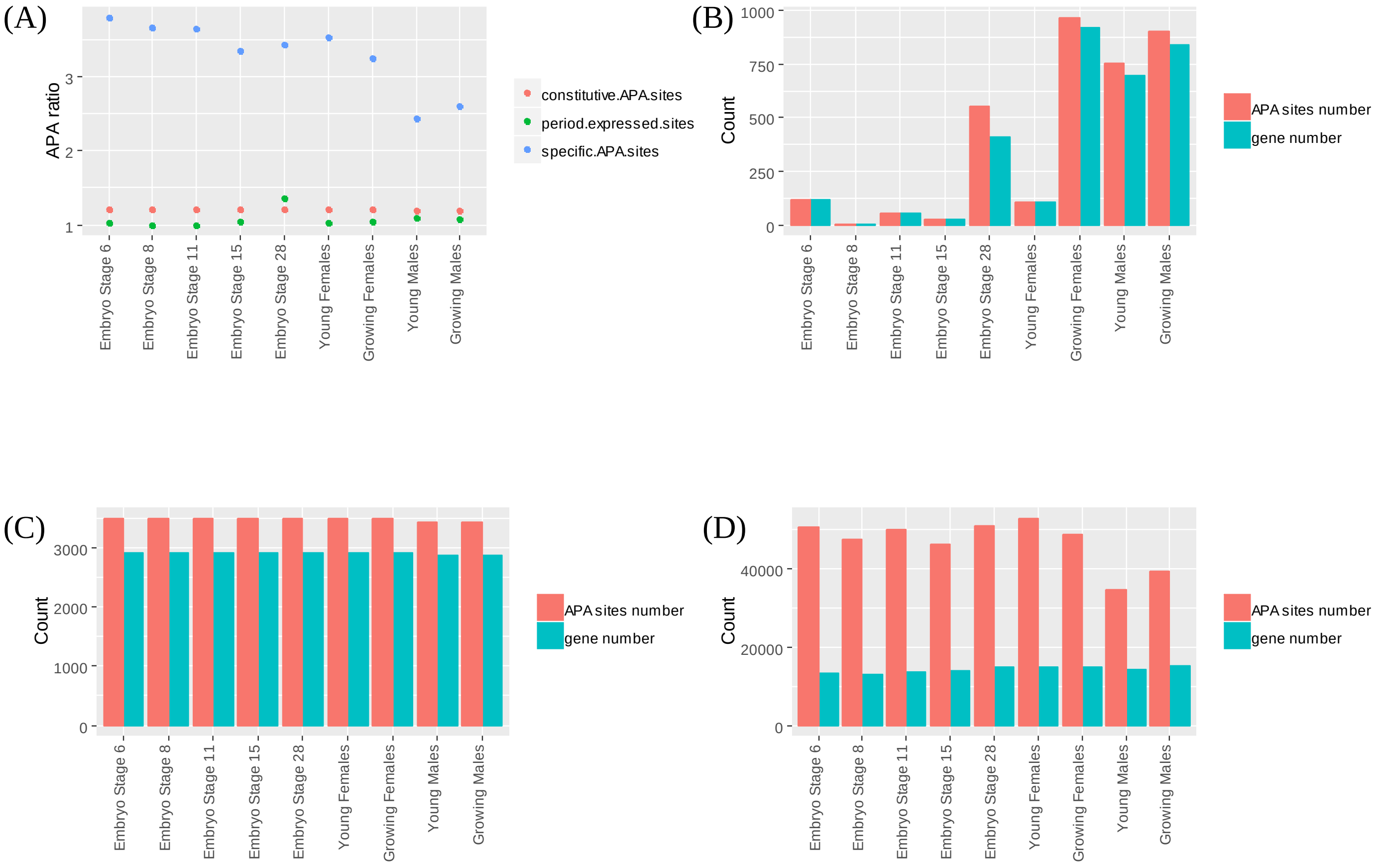

Figure S8. APA distributions and their relationships with the expressed gene number and expression level in different developmental periods. (**A**) APA ratio (APA number/gene number) of specific sites, period expressed sites, and constitutive sites. The period expressed sites refer to all poly(A) sites whose expression count is not zero at a given stage. The specific APA sites are identified according to the information entropy method. (**C**) APA number and gene number of specific sites. (**D**) APA number and gene number of constitutive sites. (**D**) APA number and gene number of period expressed sites.

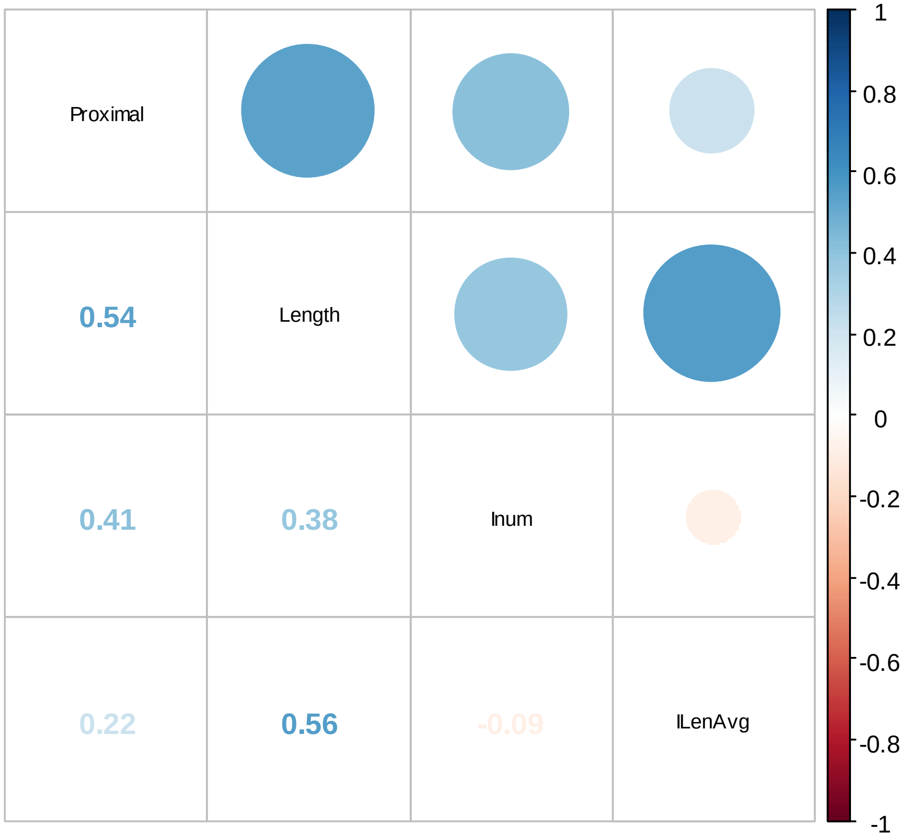

Figure S9. Investigation of the strength and direction of the linear relationship among the APA frequency in proximal regions, the gene length, the number of introns and the average length of introns. "Proximal" stands for the APA frequency in proximal regions, "Length" stands for the length of gene, "Inum" stands for the numbers of introns and "ILenAvg" stands for the average length of introns.

Table S1. Distribution of poly(A) sites.

poly(A) type	number		
poly(A) site in intergenic region	21,854(23.03%)		
poly(A) site in annotated genes	73,021 (76.97%)	protein_coding	70,918(97.12%)
		lncRNA	1,570(2.15%)
		tRNA	22(0.03%)
		other	511(0.7%)

Table S2. Distribution of poly(A) sites in different genomic regions.

Classification	Subclass	Number	Percentage (%)
Genome	protein_coding	70,918	97.12
Located in the full length cDNA	3′ UTR	21,472	30.28
	5′ UTR	3,676	5.18
	CDS	12,814	18.07
	Intron	21,483	30.29
Located nearby annotated transcript ends	Within 30nt downstream of 3′ UTR	3939	5.55
	Within 500nt downstream of 3′ UTR	6431	9.07
	Extended_3′ UTR[1]	11,394	16.07
	Within 30nt downstream of 5′ UTR	39	0.05
	Within 500nt downstream of 5′ UTR	72	0.10
	Extended_5′ UTR[2]	79	0.11

[1] Extended _3’ UTR refers to the extended region of 3′ UTR.
[2] Extended _5’ UTR refers to the extended region of 5′ UTR.

Table S3. Number of genes with different number of poly(A) sites.

		0	1	2	3	4	≥5	Total
annotated genes	Number of genes	0	3741	3020	2287	1767	5696	16511
	Percentage (%)	0	22.66	18.29	13.85	10.70	34.50	100
different region	3′ UTR	5552	5981	2450	1210	586	732	16511
	Extended_3′ UTR[1]	8548	5788	1416	463	176	120	16511
	5′ UTR	14185	1603	439	146	59	79	16511
	Extended_5′ UTR[2]	16432	79	0	0	0	0	16511
	CDS	10771	2971	1255	608	334	572	16511
	Intron	7998	3790	1953	1052	639	1079	16511

[1] Extended _3′ UTR refers to the extended region of 3′ UTR.
[2] Extended _5′ UTR refers to the extended region of 5′ UTR.

Table S4. Top 50 hexamers in upstream 100 nt to downstream 100 nt region of the 3′ UTR poly(A) sites.

Sort	Motif	Count	Percentage	Sort	Motif	Count	Percentage
1	**AAUAAA**	8390	47.73%	26	**AAAAAU**	4238	24.11%
2	**UUUUUA**	6234	35.46%	27	**AAAUGU**	4228	24.05%
3	**UAUUUU**	6222	35.40%	28	**UUAAAA**	4211	23.96%
4	**UUUAUU**	6018	34.24%	29	**UUUUUG**	4188	23.83%
5	**UUUUAU**	5990	34.08%	30	**CAUUUU**	4187	23.82%
6	**AUUUUU**	5903	33.58%	31	**GUUUUU**	4185	23.81%
7	**UUUUUU**	5833	33.18%	32	**UAUUUA**	4162	23.68%
8	**UUAUUU**	5811	33.06%	33	**AUUUUA**	4125	23.47%
9	**AAAUAA**	5705	32.46%	34	**UUUAAU**	4125	23.47%
10	**UGUUUU**	5506	31.32%	35	**AUUUAU**	4070	23.15%
11	**AUAAAA**	5416	30.81%	36	**CUUUUU**	4069	23.15%
12	**UUUUAA**	5183	29.49%	37	**UUUUCU**	4062	23.11%
13	**UUUUGU**	4984	28.35%	38	**AAAAUG**	3980	22.64%
14	**AAAAUA**	4794	27.27%	39	**UAUAUU**	3922	22.31%
15	**AUAUUU**	4792	27.26%	40	**UUUGUA**	3831	21.79%
16	**UUUAAA**	4704	26.76%	41	**UAUAAA**	3771	21.45%
17	**AUAAAU**	4692	26.69%	42	**UUAAAU**	3739	21.27%
18	**UAAAUA**	4639	26.39%	43	**UAAUAA**	3717	21.15%
19	**AUUAAA**	4596	26.15%	44	**UUUUUC**	3693	21.01%
20	**UUGUUU**	4587	26.10%	45	**AAUAUU**	3668	20.87%
21	**AAAUAU**	4502	25.61%	46	**UUUCUU**	3627	20.63%
22	**UAAAAA**	4424	25.17%	47	**UGUAUU**	3625	20.62%
23	**UAAAAU**	4375	24.89%	48	**AUGUUU**	3604	20.50%
24	**UUUGUU**	4360	24.80%	49	**UUAUAU**	3559	20.25%
25	**UUUAUA**	4280	24.35%	50	**AUAUAU**	3534	20.10%

Table S5. T-test for the difference of the U content on DSE among the five types of sites.

strong sites	1				
universal sites	3×10^{-5}	1			
unique sites	0.28	3×10^{-4}	1		
weak sites	3×10^{-5}	1	3×10^{-4}	1	
control sites	2×10^{-18}	3×10^{-20}	2×10^{-19}	4×10^{-20}	1
p-value	strong sites	universal sites	unique sites	weak sites	control sites

Table S6. T-test for the difference of the U content on USE among the five types of sites.

strong sites	1				
universal sites	0.08	1			
unique sites	0.75	0.13	1		
weak sites	0.08	0.10	0.13	1	
control sites	2×10^{-6}	3×10^{-5}	2×10^{-6}	3×10^{-5}	1
p-value	strong sites	universal sites	unique sites	weak sites	control sites

Table S7. T-test for the difference of the A content in the region of -34 nt to -13 nt among the the five types of sites.

strong sites	1				
universal sites	0.02	1			
unique sites	0.78	0.04	1		
weak sites	0.02	1	0.04	1	
control sites	4×10^{-9}	9×10^{-10}	1×10^{-9}	9×10^{-10}	1
p-value	strong sites	universal sites	unique sites	weak sites	control sites

Table S8. Signal patterns and consensus sequences identified for poly(A) sites in different genomic regions.

region	signal element			sequence	observed occurrences	expected occurrences	p-value	occurrence significance
CDS	USE		significant patterns	UCAUCA	1063	513.64	1.30E-99	95.58
				CUCCUC	1245	657.6	1.90E-92	88.41
				GAAGAA	1944	1237.32	5.40E-77	72.95
			overlapping patterns	AAGAAA	2331	1749.56	3.00E-40	36.21
			consensus sequences	AUCAUCAU, CCUCCUCC				
	PE		significant patterns					
			overlapping patterns					
			consensus sequences					
	CE		significant patterns	ACUUAC	680	232.61	6.00E-125	120.88
				AAGGUA	1527	807.75	2.00E-112	108.45
				AUUUUC	962	438.3	2.00E-103	99.39
			overlapping patterns	AAGAAA	3486	2920.75	1.40E-24	20.52
			consensus sequences	ACUUACCUUUU, CCUCCUCC, AAGAAGAA				
	DSE		significant patterns	CUCCUC	592	310.84	9.50E-46	41.7
				AGGUAA	684	378.34	2.20E-43	41.35
				ACUUAC	543	279.76	2.80E-44	40.23
			overlapping patterns	AAAAUA	1262	1004.05	2.60E-15	11.27
			consensus sequences	CCUCCUCC, CAGGUAAGU, ACUCACCU				
intron	USE		significant patterns	AAAUAA	2506	1773.11	1.20E-60	56.6
				AAUAAA	2503	1773.11	3.40E-60	56.15
				GAAGAA	1199	825.93	2.90E-34	30.22
			overlapping patterns	AAAAUA	2574	2180.03	1.20E-16	12.62
			consensus sequences	UAAAUAAA, CCUUCCU, AAAUGAAGAAGAG				
	PE		significant patterns	AAUAAA	3954	1729.54	0	350
				AAAUAA	2903	1729.54	1.00E-146	142.66
				UAAUAA	1527	759	7.00E-133	128.83
			overlapping patterns	AAUAAA	3954	1729.54	0	350
			consensus sequences	AAAUAAA, A-rich				
	CE	CEL	significant patterns	CAGUAG	320	60.71	8.00E-121	116.76
				AGUAGG	305	55.1	2.00E-120	116.47
				CCCUAC	205	40.43	2.70E-75	71.26
			overlapping patterns	AAAAAG	648	493.43	1.60E-11	7.48
			consensus sequences	ACAGUAGGGCAA, ACUUACCUU, ACAGUAGGACAA				
		CER	significant patterns	GGAGAC	587	157.63	4.00E-151	147.11
				GAUGGA	625	182.09	7.00E-145	140.81
				GAGACA	756	286.12	2.00E-117	113.35
			overlapping patterns	AAUAAA	1461	1159.29	8.30E-18	13.76
			consensus sequences	AGGGCAAGAUGGAGACAGUAGGGC				
	DSE		significant patterns	AAUAAA	2633	2813.86	9.40E-50	45.71
				CCUUUA	1635	1138.23	1.10E-43	39.63
				GAGAGA	922	571.48	1.70E-41	37.46
			overlapping patterns	AAUAAA	2633	2813.86	9.40E-50	45.71
			consensus sequences	AUAAAUAAA, UCCUUCCUUUA, AGAGAGAAGA				
5' UTR	USE		significant patterns	GAGAGA	326	183.31	1.40E-21	17.54
				AGGAAG	274	149.96	7.10E-20	15.83
				AAUAAA	545	367.66	3.40E-18	14.15
			overlapping patterns	AAUAAA	545	367.66	3.40E-18	14.15
			consensus sequences	AGAGAGA, AAGGAAGGA, UAAAUAAA				
	PE		significant patterns	AAUAAA	587	242.15	1.00E-78	74.68
				AGAUAA	81	20.07	1.40E-24	20.55
				CAAUAA	150	59.07	7.90E-23	18.79
			overlapping patterns	AAUAAA	587	242.15	1.00E-78	74.68
			consensus sequences	CAAUAAAUAA, AGGAAGAUGGAG, UUUAAUAAAUAA				
	CE		significant patterns	AGAUGG	72	25.77	6.30E-14	9.88
				CAGUAG	61	19.68	7.00E-14	9.84
				AAAUGA	130	63.7	2.10E-13	9.35
			overlapping patterns	AAAGGA	184	110.13	7.90E-11	6.79
			consensus sequences	CAAGAUGGAG, CAGUAGGGC, AGAAAGGAAAAUGAU				
	DSE		significant patterns	GAGAGA	338	203.06	3.30E-18	14.16
				AGCAGC	323	192.77	7.50E-18	13.81
				GAAGAA	248	146	1.00E-14	10.67
			overlapping patterns	AAUAAA	594	439.04	1.20E-12	8.59
			consensus sequences	GAGGAGAGACA, AGCAGCC, AAUG				
